# Advances in waveguide to waveguide couplers for 3D integrated photonic packaging

**DOI:** 10.1038/s41377-025-02048-w

**Published:** 2026-01-01

**Authors:** Drew Weninger, Samuel Serna, Luigi Ranno, Lionel Kimerling, Anuradha Agarwal

**Affiliations:** 1 Materials Research Laboratory, Massachusetts Institute of Technology, 77 Massachusetts Ave, Cambridge, 02139 MA USA; 2 Department of Physics, Photonics, and Optical Engineering, Bridgewater State University, 131 Summer St, Bridgewater, 02324 MA USA; 3 Department of Materials Science and Engineering, Massachusetts Institute of Technology, 77 Massachusetts Ave, Cambridge, 02139 MA USA; 4 Present Address: Physical Measurement Laboratory, National Institute of Standards and Technology, 100 Bureau Dr, Gaithersburg, 20899 MD USA; 5 Present Address: Ayar Labs, 695 River Oaks Pkwy, San Jose, 95134 CA USA

**Keywords:** Integrated optics, Silicon photonics, Fibre optics and optical communications, Micro-optics

## Abstract

In this paper, we provide an overview and comparison of devices used for optical waveguide-to-waveguide coupling including inter-chip edge couplers, grating couplers, free form couplers, evanescent couplers, cantilever couplers, and optical wirebonds. In addition, technology for efficient transmission of light through chips is discussed including guided mode and free form photonic vias for substrates including silicon, glass, and organics. The results are discussed in the context of potential applications including co-packaged optics switch packages, replaceable biochemical sensors, optically connected memory, optical computing, integrated quantum photonics, and integrated LiDAR systems to show possible improvements in energy efficiency, performance, and cost.

## Abstract (web summary)

The automated packaging and assembly of a photonic chiplet to an optical interposer and printed circuit board is shown, where optical inter-chip couplers, wirebonds, and vias provide 3D waveguide connectivity.

## Graphical abstract

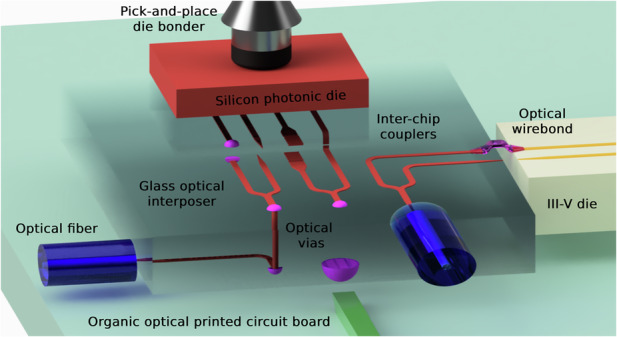

## Introduction

**Fig. 1:**
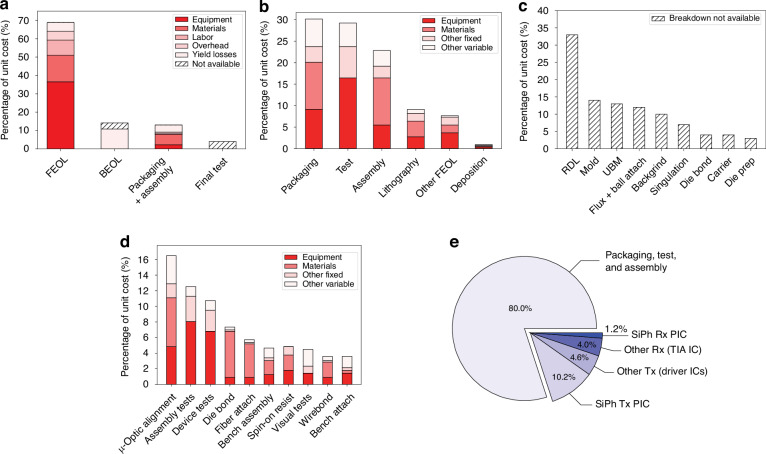
Cost breakdown for packaging in microelectronics and microphotonics. In (**a**), data for Samsung’s fan-out panel level packaging (FO-PLP) manufacturing of the Google Tensor G2 system-on-chip (SoC)^11^. The data in (**b**) shows similar data for the manufacturing of a fiber coupled, integrated InP PIC with modulators and detectors^12^. The data in (**c**) is for FO-WLP, but nonetheless provides information on a breakdown by process for electronic packaging cost drivers^13^. Similarly, (**d**) does the same breakdown by process for the InP PIC^12^. Finally, (**e**) provides the overall cost breakdown for a ubiquitous electronic-photonic system—a SiPh transceiver^8^

Photonic integrated circuits (PICs) have seen significant advancement over the past 40 years, highlighted by their rise to dominance in data center interconnects^1^ and the novel application of PICs in biochemical sensors^2^, light detection and ranging (LiDAR)^3^, photonic switching^4^, photonic computing^5^, and even chip based 3D printing^6^. While the high volume manufacturing of PICs has progressed significantly, evidenced by the fact that 10^3^–10^5^ devices can be integrated onto a single die^1^, their packaging, assembly, and testing has not. Common estimates hold PIC packaging, assembly, and testing responsible for 70−80% of the total cost of PIC manufacturing compared with only 20% for their electronic integrated circuit counterparts^7–10^ as shown in Fig. 1. Specifically, the data in Fig. 1a shows the breakdown for a state-of-the-art electronic system-on-chip (SoC), demonstrating that front-end-of-line (FEOL) and back-end-of-line (BEOL) fabrication processes in foundries dominates the spending of chip manufacturing^11^. Meanwhile, the data in Fig. 1b for PICs—in this case indium phosphide (InP)^12^—shows the situation reversed. Furthermore, the costs can be broken down by process as in Fig. 1c showing the most expensive processes for electronic fan-out wafer-level-packaging (FO-WLP)^13^, which is contrasted by Fig. 1d showing the percentage of total cost for packaging, assembly, and test processes in integrated photonics^12^. This discrepancy is not simply due to electronics die being substantially more expensive than photonics die overall—in fact, relatively simple PICs cost roughly equivalent to the most advanced electronic ICs. For example, a state-of-the-art electronic SoC fabricated on a 5 nm process node costs approximately $0.57 per mm^2^ ^14^ to purchase from a fabless design firm before packaging, while a SiPh^15^ or InP PIC costs approximately $0.1–0.4 per mm^2^ ^16^, respectively, before packaging for chip volumes in the several millions. This is reflected by the fact that when we look at an example electronic-photonic system, such a silicon photonic (SiPh) pluggable transceiver, the packaging, assembly, and testing still dominates the overall cost in comparison to the SiPh transmitter (Tx) or receiver (Rx) die and the required electronics die such as transimpedance amplifiers (TIAs) or other drivers^8^ as shown in Fig. 1e.

### Current techniques in semiconductor packaging and assembly

#### Electronic packaging and assembly strategies

**Fig. 2:**
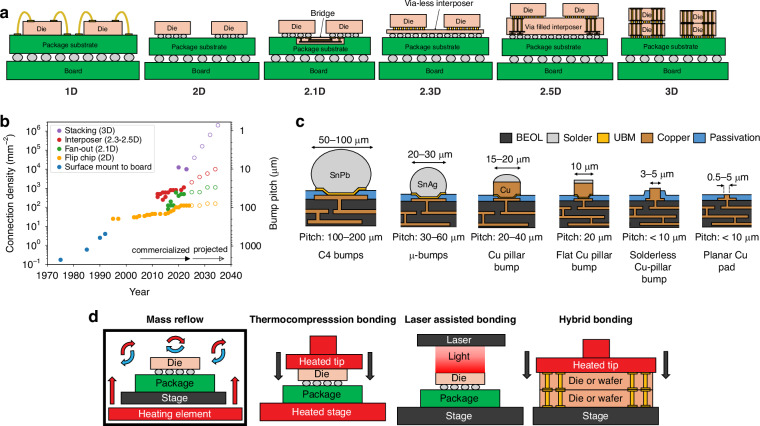
Figures showing common electronic packaging and assembly techniques. In (**a**), the evolution from wirebonding (1D) to stacked die (3D) shows the increasing complexity of wiring within packages^368^. The plot in (**b**) shows the progression of bump technology over time (data up to 2020 is from ref. ^369^, with data after 2020 and future projections from refs. ^30,370^ which was in part derived from ref. ^371^), while (**c**) shows cross sections of the bumps used today, with both figures demonstrating current bumping pitches of <10 μm are possible^370^. The images in (**d**) show the common bonding processes used during assembly, which depends on the bumps technology used

Understanding the current technology landscape in electronic and photonic packaging can help provide answers to as to why photonic packaging and assembly continues to prove a significant challenge. Starting in 1947 with the first discrete wire connection^17^ in the first transistor^18^ and continuing with the development of ball and wedge bonding in the 1950s by Bell Labs^19^, electrical components have been connected using gold (Au), aluminum (Al), or copper (Cu) based wirebonds in a serial process. Today, electrical wirebonds (a 1D architecture using the nomenclature from ref. ^20^) can be made with 35-μm pitch^21,22^ and a time per bond of ~0.1 s^23^ using automated tools^24,25^, and remain the most ubiquitous interconnect modality for applications requiring <10^3^ connections. In the 1960s, IBM proposed^26^ and introduced^27^ the first parallel process for electrical interconnection with the advent of the flip chip solder connection (a 2D architecture). Specifically, ball grid arrays (BGAs) using solder bumps coined controlled-collapsed-chip-connections (C4 bumps)^28^ enabled multiple connections from a die to a printed circuit board (PCB) in a single bonding step. Over time, in order to effectively scale the number of connections made to the chip, flip chip packaging strategies became more advanced with the number of intermediate substrates scaling up, as in Fig. 2a, and the solder bump diameters scaling down, as in Fig. 2b, c. Thus, for high volume applications requiring >10^3^ electrical connections to be made, flip chip technologies are typically used.

Currently, advanced electronic packages are typically composed of an advanced node electronic die bonded to a redistribution layer (RDL) or embedded bridge (called a 2.1D architecture) which is fabricated on an organic package substrate, or a die bonded to a silicon interposer which is bonded to the organic package substrate (termed 2.3D or 2.5D architecture depending on if the interposer is thick enough to have electrical vias or not)^29^. Typically, the purpose of adding RDLs, bridges, or interposers is to achieve a higher level of electrical fan-out within the package—these components contain no functional devices on them, only Cu traces or vias. The fan-out itself is achieved by scaling the bump technology as one goes higher into the package stack, with Cu pillar bumps at the die-to-interposer level having pitches <20 μm compared to the C4 connections between the package substrate and the board having >100 μm pitch^30^. Stacking of electrical die atop one another (a fully 3D architecture), a practice common in the packaging of advanced dynamic random access memory (DRAM) chips^31^ and complimentary metal-oxide-semiconductor (CMOS) image sensors^32^, is also possible. These 3D architectures target connection densities >10^4^ connections per mm^2^ and bump pitches <10 μm by using through-silicon-vias (TSVs) combined with hybrid bonding^33^, which will be described below.

The processes used in the assembly and bonding of the above architectures can be grouped into four categories: mass reflow, thermocompression bonding (TCB), laser assisted bonding (LAB), and hybrid bonding. Cross sectional schematics showing the basic operation of each process can be found in Fig. 2d. The type of bonding process used depends on the type of bump used. For example, mass reflow, where die are assembled onto substrates using high speed, automated pick-and-place tools and then collectively heated in a reflow oven such that the C4 bumps melt and solidify, is often used in the case of C4 bumps where the pick-and-place alignment can be quite poor and still result in high yield. On the other hand, TCB, where pressure and heat are applied to the die during the pick-and-place step, is used with *μ*-bumps or Cu pillars where higher alignment accuracy is needed along with pressure in order to prevent or remove oxide films on the electrical bumps which lead to electrical failure. Because TCB is a serial process where one die is bonded after the next, the lower throughput^34^ for such processes is often seen as a substantial downside. As mentioned above, hybrid bonding is used with planarized, solderless Cu/dielectric interfaces to achieve the highest possible connection density. Hybrid bonding involves taking a surface composed of Cu pads and dielectric passivation (such as silicon dioxide (SiO_2_), silicon oxynitride (SiON), or silicon carbonitride (SiCN)), planarizing it using chemical mechanical polishing (CMP), activating the surface by applying plasma, and then bonding the dielectric surfaces and Cu surfaces such that the entire surface of each die is part of the bonding interface^35^. The hybrid bonding process can be done at the die level or wafer level, although the ability to determine known-good-die (KGD) prior to bonding led to the development of collective die-to-wafer (D2W) bonding^36^ (also called advanced chip-to-wafer bonding)^37^. Collective D2W bonding increases throughput while allowing for determination of KGD by performing high speed pick-and-place of die onto a carrier wafer bonded using a sacrificial bond material (a low time, low temperature process), and then collectively bonding all of the die on the carrier wafer to the target wafer in a single bonding step (a single long, high temperature process).

Of critical importance to each of the above bonding and bumping technologies is the role of the automated pick-and-place die and wafer bonders. These advanced tools allow for high accuracy die or wafer alignment simultaneously with high speed, and are often equipped with additional capabilities such as epoxy dispense, heated tips and stages, and ultraviolet (UV) curing lamps. As one might expect, there is a tradeoff between speed (i.e., throughput) and alignment accuracy on these tools, which becomes relevant when moving to increasingly smaller bump pitches. Standard pick-and-place tools used for die attach prior to mass reflow have alignment accuracies of 10–20 μm and throughputs upwards of 18,000 units per hour (UPH)^38^, with higher accuracy models being available for placement precisions <3 μm with speeds of >2000 UPH (assuming 1–2 s per bond)^34^ for TCB^39,40^, and the highest accuracy die bonders being able to align to <0.3 μm with speeds of 2000 UPH for hybrid bonding^41^. Wafer bonders usually offer an order of magnitude better alignment accuracy than die bonders, with tools offering precisions <50 nm for 300 mm wafers^42^ and post-bond accuracies of <150 nm shown experimentally for Cu–Cu interfaces^43^. At the research stage, firms are also moving towards panel level bonders in order to enable the full potential of substrates such as glass or organics which can be fabricated at the panel level^44^. For electronics, the combination of methods such as collective D2W or panel level assembly with hybrid bonding in 3D architectures, which has shown to be possible with <2 μm final accuracy^45^, could prove critical to continued exponential scaling of performance in the most advanced systems. However, the application of these advanced flip chip packaging techniques to photonics could prove to have an equal, or potentially larger, impact on future electronic-photonic systems.

#### Photonic packaging and assembly strategies

**Fig. 3:**
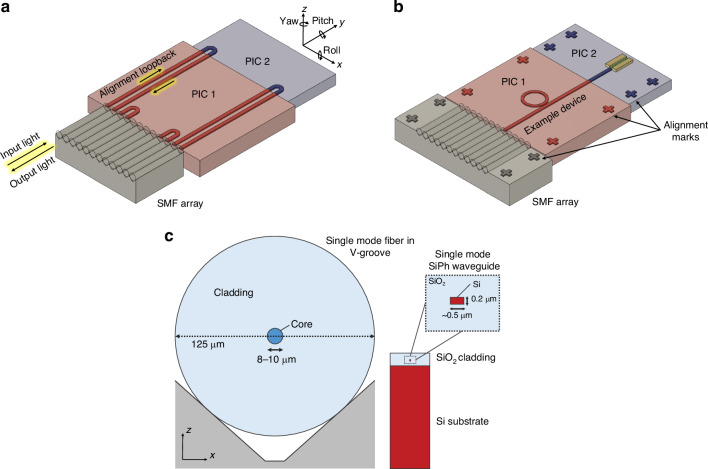
Alignment techniques in fiber-to-chip assembly in integrated photonics. In (**a**), active alignment of fiber to chip, or chip to chip, is shown with the required alignment loopbacks. In (**b**), passive assembly using only machine vision with alignment features is shown. In (**c**), a standard SMF in a V-groove array is shown compared to a standard single mode SOI waveguide

One of the primary barriers to cost-effective photonic packaging and assembly is the active alignment and bonding of single mode fiber (SMF) arrays to PICs using UV-curable epoxies in a serial fashion, increasing cost and limiting throughput. Active alignment is the process of bringing a fiber array (or die) close to another die, injecting light through the fiber, and constantly scanning the position of the fiber array until maximum output power is measured through a second fiber in the array connected using a loopback, as shown in Fig. 3a. Once the maximum output power is measured, the fiber array is typically bonded into position using UV-curable epoxies, and the fiber position must be actively maintained and manipulated during the curing process as the epoxy will shrink or expand while solidifying. The technique of active alignment is currently the accepted method for fiber-to-chip attach or chip-to-chip attach in integrated photonics^46^. This is contrast to passive alignment, the method ubiquitously used by high speed pick-and-place die bonders of electronic packaging, which relies only on locating alignment features (fiducials) to determine where the center of a component is, as shown in Fig. 3b, and then placing that component at a given coordinate^47^. Not only are current active alignment based packaging methods costly in photonics, but they are severely limited in terms of their ability to scale optical input/output (I/O), or the number of connections made to the PIC. For example, SMF arrays operating near datacom (1310 nm) or telecom (1550 nm) wavelengths have minimum pitches of 127 μm due to their 125 μm cladding as shown in Fig. 3c, meaning a maximum density of only 8 fibers per mm is possible. To add to this challenge, as shown in Fig. 3a, multiple input and output ports to the die must be consumed with alignment loopbacks—waveguides and fibers which serve no other purpose other than to ensure fiber attach can be accomplished.

**Fig. 4:**
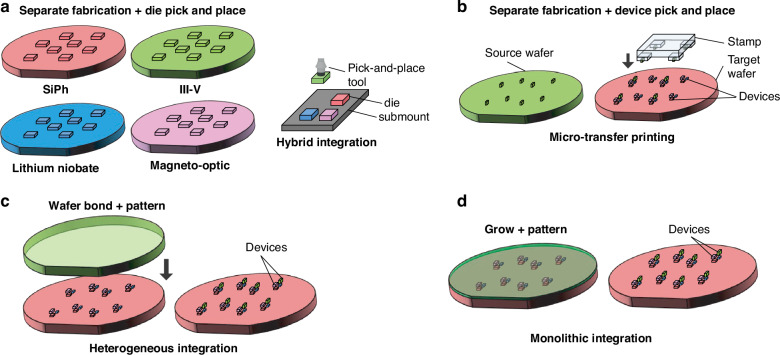
Techniques for integrating separate material platforms in integrated photonics. In (**a**), the hybrid bonding of PICs from a few example material platforms is shown. The devices from those different material platforms can also be integrated using other pick-and-place methods such as *μ*-TP in (**b**). The devices can also be integrated in the FEOL using wafer bonding (i.e., heterogeneous integration) as in (**c**) or epitaxial growth (i.e., monolithic integration) as in (**d**)

In addition to the fiber attach process, another photonic packaging challenge is the integration of devices from separate materials into a single package or across a single die. Four primary techniques have been developed to address this as outlined in Fig. 4: hybrid integration^48^, micro-transfer printing (*μ*-TP)^49^, heterogeneous integration^50,51^, and monolithic integration^52^. The four strategies each have tradeoffs and vary in terms of their level of integration and maturity. For instance, hybrid integration (shown in Fig. 4a) fabricates die separately in optimized foundry process flows and then uses pick-and-place of die on a submount for connectivity. The process of *μ*-TP, shown in Fig. 4b and still in the proof-of-concept stage, operates similar to hybrid integration, except the pick-and-place happens at the device level using a specialized plastic stamp. Thus, devices are ultimately transferred across the entire target wafer with only a few pick-and-place steps. The processes offering higher levels of integration include wafer bonding (heterogeneous integration, shown in Fig. 4c) or epitaxially growing (monolithic integration, shown in Fig. 4d), or combining the two^53^, the new material and then performing standard lithographic patterning thereafter. While these techniques offer higher levels of integration, they present difficulties in terms of the co-processing of the SiPh and the non-native components and the ability to test or inspect the non-native components for KGD.

For solutions to the challenge of fiber attach and material integration, one needs only to look to microelectronics packaging where cost-effective I/O scaling is achieved using wirebonding or flip-chip connections depending on the number of connections needed. The development of optical equivalents to wirebonds, flip-chip connections, and electrical vias has therefore become a fervent research area, with the goal of improving the cost, size, weight, and power (C-SWaP) in photonic packages. As this research area is currently in its infancy, these optical equivalents are being developed without standards in terms of materials, processes, and performance metrics. To this end, it is useful to compile a database of available optical devices which can provide high-efficiency inter-chip connections. The term “inter-chip” refers to coupling from a single mode waveguide on a PIC (also referred to as a die or chip), interposer, or board to a single mode waveguide on a separate die, interposer, or board. Similarly, “intra-chip” refers to connections made between different layers of the same chip, and designs meant for intra-chip coupling through the bulk of a chip are referred to as photonic vias. In general, inter-chip optical coupling present substantial challenges versus intra-chip coupling such as needing to couple over >1 μm distances without high-resolution inter-chip alignment tools and difficulty associated with routing light out-of-plane. However, continued advancements in automated, pick-and-place die bonder resolution have demonstrated sub-micron placement accuracies with high speed using commercially available equipment^54,55^. Other bonding methods have evolved as well, including wafer scale processes such as collective die-to-wafer hybrid bonding^56^ or micro-transfer printing of thick components^49,57^. Novel fabrication techniques for creating on-chip 3D micro-optical components such as grayscale lithography (GSL)^58–62^, two-photon polymerization (TPP) lithography^23^, and surface controllable refractive index via beam exposure (SCRIBE)^63,64^ have unlocked integration possibilities not previously considered. Thus, with the need for optical I/O densities >8 connections/mm and C-SWaP scaling for ever more complex integrated photonic systems, inter-chip optical couplers have become a critical enabler for future PIC applications from the visible to the mid-infrared (mid-IR).

The design of these inter-chip connections would ideally meet the requirements laid out in Table 1 to be useful in practical applications and provide an improvement over fiber-to-chip packaging. These performance metrics will serve as the basis of comparison for these inter-chip couplers and a starting point for future comparisons. The following section discusses different types of inter-chip optical couplers and their operation.

**Table 1:** Performance metrics and associated requirements for inter-chip optical couplers

Metric	Definition	Units	Requirement	Benefit of meeting requirement
Insertion loss	Minimum loss when couplers are in optimized position	dB^a^	<1 dB	Improves energy efficiency
1-dB alignment tolerance	Misalignment allowed before 1 dB of excess loss	μm	>±1–10 μm >±0.4° twist	Enables passive assembly using pick-and-place tools
Foundry compatibility	Ability to be integrated into Si-CMOS process flows	/	Limited BEOL customization	Cost reduction and scalability
Throughput	Ability to use parallel versus batch versus serial processes	/	Parallel processes	Cost reduction and scalability
Footprint	Dimensions (L × W) of the coupling element occupying the most space	μm	<<1000 μm (L) <<125 μm (W)	Increases connection density
Pitch	Minimum distance required between couplers before crosstalk occurs	μm	<<125 μm	Increases connection density
Channel density	Inverse of pitch for a given direction	couplers/mm	>>8 couplers/mm	Increases connection density
1-dB bandwidth	Change in wavelength before 1 dB excess loss occurs	nm	>100 nm	Supports WDM functionality (different optimized wavelength supports novel applications)
Polarization dependent loss (PDL)	Loss penalty incurred when operating with TM versus TE mode (and vice versa)	dB	<0.5 dB	Supports polarization based multiplexing
Temperature tolerance	Change in temperature before 1 dB excess loss occurs	°C	>100 °C	Supports operation in thermally challenging environments such as space or data centers

*WDM* wavelength division multiplexing, *TM* transverse magnetic, *TE* transverse electric

^a^Note that $loss[dB]=-10{\log }_{10}(\eta )$ where *η* is coupling efficiency

## Types of waveguide to waveguide couplers

**Fig. 5:**
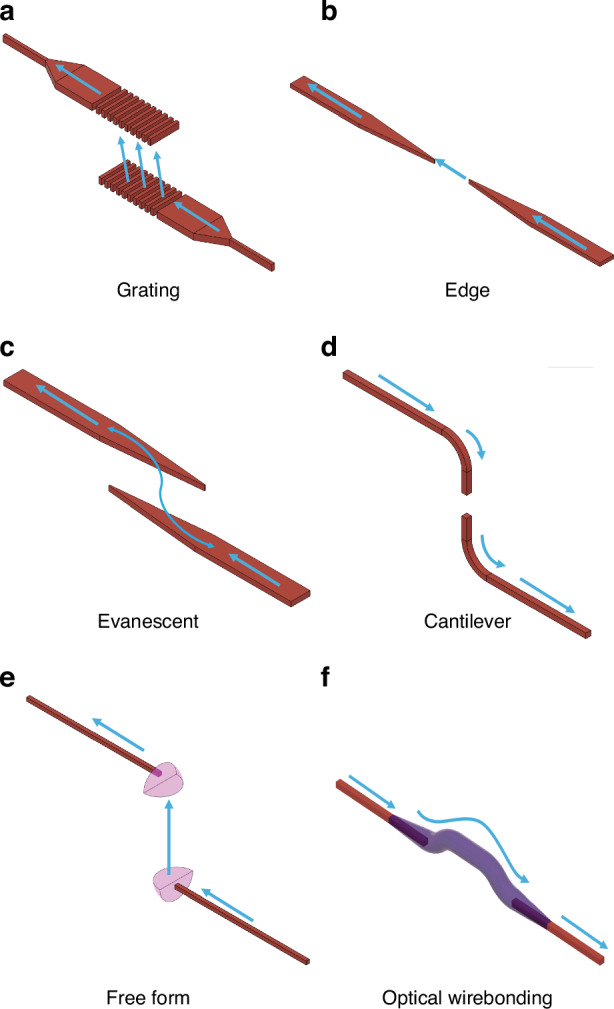
General inter-chip coupling architectures. The images each show an example scenario of coupling between SOI waveguides (shown in red) on two separately fabricated chips, with (**a**) representing grating-to-grating coupling, (**b**) edge coupling, (**c**) evanescent coupling, (**d**) cantilever coupling, (**e**) free form coupling, and (**f**) optical wire bonding. The blue arrows indicate the direction of optical propagation before, during, and after coupling. Any pink structures in the images indicate components which are typically of a different material composition from the waveguides

**Fig. 6:**
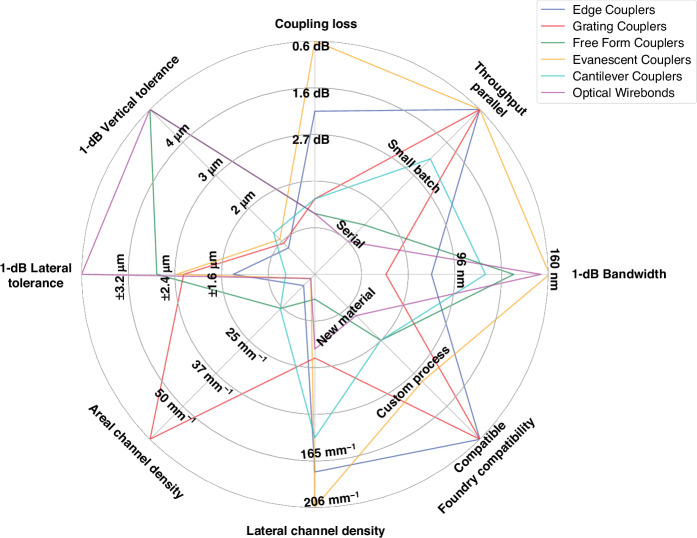
A comparison of different types of waveguide-to-waveguide coupling schemes in terms of several figures of merit. The solid lines plotted for each coupler category represent averages for each figure of merit. This data was compiled based on the values reported Tables 2–8 of this study, with the exception of throughput and foundry compatibility. Specifically, the following references were used for each coupling class: edge^75,76,78,79,81–83,88,89,91,163,322^, grating^100–102,124,126–128,323–332^, free form^58,59,130,131,133,136,140,207,220,221,333–339^, evanescent^9,50,56,153–156,162,163,244,249,253,289,340–363^, cantilever^164–166^, and optical wirebonds^64,163,175–185,193,364^

The optical coupling designs proposed in literature for inter-chip coupling can be categorized into six different types: edge, grating, free form, evanescent, cantilever, and optical wirebonds. Schematics showing the general operation of these six types can be found in Fig. 5. Additionally, Fig. 6 shows values for each figure of merit, and for each coupling type, as defined in “Introduction”. The values for each figure of merit shown in Fig. 6 are averages taken over each data point in Tables 2–8. For the metrics of throughput and foundry compatibility, each coupling type was qualitatively assessed based on the fabrication, packaging, and assembly processes used (e.g., standard 193/248 nm deep UV (DUV) photolithography versus custom two-photon polymerization) and the materials required. The plot in Fig. 6 can be used as a guide throughout the following sections showing how coupling types compare against one another and which may be best suited for different applications. The remaining sections describe the operation of each coupling type, provide examples of notable designs and results, and present tables quantitatively comparing each coupler that was reviewed.

**Table 2:** Summary of inter-chip edge coupler devices

Package	Design	IL (dB)	Pol.	1-dB Toler. (μm)			1-dB BW (nm)	L × W × H (μm)	Thru.	Process	Ref.
				Lateral	Vertical	Long.					
SOI to SOI^a^	H-bar MMI	0.32 0.56	TE TM	/	/	/	>40(1530–1570)	241 × 2 × 5	Parallel	Custom	^88^
Ge-on-Si to Ge-on-Si	OCQ	4.1	/	**±2**	±1.25	0.5	/ (8400)	16 × 12 × 4	Parallel	Custom	^81–83^
III-V to SOI	Trident taper	2	/	±1.4	±0.35	1.4	60 (1490–1550)	96 × 9.6 × 0.22	Parallel	Standard	^79^
InP to SOI	Inverse taper	0.25	/	**±0.7**	**±0.5**	/	120 (1510–1630)	75 × 0.4	Parallel	Standard	^76^
InP to SOI	Meta-material	0.9	/	**±1.7**	**±0.5**	/	120 (1510–1630)	250 × 0.4	Parallel	Standard	^76^
Polymer to polymer^a^	MMI	1.0	/	**±2**	/	/	90 (1510–1600)	173 × 4 × 10.4	Parallel	New	^89^
Si_3_N_4_ to ULI	Inverse taper	2.8	/	±1.5	±1.8	/	/ (1550)	200 (L)	Serial	New	^163^
InP to SOI	Inverse taper	1.6	/	±1.5	±1	/	45 (1515–1560)	600 × 3	Parallel	Standard	^78^
**GaSb to SOI**	**Angled facet & taper**	**1.46**	**TE**	**>±1**	**±0.13**	**0.75**	**/ (1881–1947)**	**200** **×** **6** **×** **1**	**Parallel**	**Custom**	^85^
GaSb to SOI	Angled facet & taper	2.7	TE	/	/	/	/ (1955–1992)	200 × 6 × 1	Parallel	Custom	^84^
**GaSb to SOI**	**Angled facet**	**1.8**	**TE**	**±0.45**	**±0.97**	**1.46**	**/ (2000)**	**5** **×** **3(W** **×** **H)**	**Parallel**	**Standard**	^48^
InP to SiN	Inverse taper	**1.5**	/	±0.5	±0.4	1.1	/ (1550)	2.15 (W)	Parallel	Standard	^75^
Polymer to polymer^a^	MMI	7.05	/	/	/	/	/ (1550)	486 × 18 × 18	Parallel	New	^91^
**SiN**_***x***_ **to SiN**_***x***_	**GRIN**	**0.33** **0.41**	**TE** **TM**	**±2.38**	**±2.24 11(rise)**	**/**	**>360(1280–1640)**	**66** **×** **11** **×** **11**	**Parallel**	**Custom**	^322^
**SOI to SOI**	**GRIN**	**0.35** **0.43**	**TE** **TM**	**±2.38**	**±2.24 11(rise)**	**/**	**>360(1280–1640)**	**66** **×** **11** **×** **11**	**Parallel**	**Custom**	^322^
**GaSb to SOI**	**Angled facet**	**1.94**	/	/	/	**1.59**^b^	**/ (2000)**	**3.1** **×** **5.4(W** **×** **H)**	**Parallel**	**New**	^87^
Polymer to polymer^a^	MMI	2.4	/	± 0.5^c^	23(rise)	/	40 (1300–1340)	3223 × 7.7 × 22.5	Parallel	New	^93^

The bold values indicate that only simulation results were reported

*BW* bandwidth, / not reported

^a^Intra-chip coupling

^b^Estimated based on reported simulation data

^c^Offset from MMI core to waveguides

**Table 3:** Summary of inter-chip grating coupler devices

Package	Period (nm)	IL (dB)	Pol.	1-dB Toler. (μm)		1-dB BW (nm)	L × W (μm)	Thru.	Process	Ref.
				Lateral	Vertical					
SOI to SOI	586	2.8	/	± 2	/	± 15 (1543)	40 × 25	Parallel	Standard	^323^
SOI to SOI	632	6.0	/	±3	/	± 40 (1550)	10 × 10^a^	Parallel	Standard	^101^
SOI to SOI	630	8.0	/	±1.5	± 0.2	<± 6 (1550)	10 × 10	Parallel	Standard	^324^
SOI to SOI^b^	820 (along) 390 (lateral)	6.02	/	/	/	±5 (1560)	20.5 × 13	Parallel	Custom	^325,326^
SOI to SiN^b^	600 (Si) 1000 (SiN)	2.03	TE	**± 0.25**	± 0.1	<10 (1470)	12 × 18	Parallel	Custom	^126^
SiN to SiN	930	**3.87**	/	/	/	**±100 (1550)**	25.79 × 25.79	Parallel	Standard	^124^
SOI to SOI	/	4.0	/	± 2	/	< ± 10 (1310)	/	Parallel	Standard	^100^
a-Si to a-Si^b^	640	0.81	TE	/	**>4**	30 (1570–1600)	12 × 5	Parallel	Custom	^127,327–331^
SOI to SOI	/	**5.9**	/	**± 2.25**	**±1**	< ± 7 (1505)	/	Parallel	Standard	^332^
SOI to SOI (no reflector)	534	**4.8**	/	**± 2.44**	**<0.25**	**± 42.5 (1310)**	15 × 15	Parallel	Standard	^128^
SOI to SOI (reflector)		**0.4**		**± 4.53**	**<0.1**	**± 17.5 (1310)**			New	
SOI to SOI	528	0.94	/	/	/	21 (1539–1560)	20 × 20	Parallel	Standard	^102^

The bold values indicate that only simulation results were reported

*BW* bandwidth, / not reported

^a^Estimated based on reported MFD

^b^Intra-chip coupling

**Table 4:** Summary of inter-chip free form couplers

Package	Design	IL (dB)	Pol.	1-dB Toler. (μm)			1-dB BW (nm)	L × W × H (μm)	Thru.	Process	Ref.
				L (μm)	V (μm)	*∠* (°)					
InP to SMF	Wet etch mirror	8.6 11.3	TE TM	/	/	/	< ± 0.5 (1550)	12 × 15 × 5	Parallel	Custom	^333,334^
InP to SMF	FIB etch mirror	< 9	/	/	/	/	/	4 (W)	Serial	Custom	^133^
**InP to SOI**	**Mirror & grating**	**0.71**	/	**±** **2**	**±** **1**	/	**80 (1505–1585)**	/	**Parallel**	**Custom**	^335–337^
SiO_*x*_ to polymer	Mirror & lens	2.6^a^	TE TM	± 1.5	± 5	/	100 (1500–1600)	24 (L)	Serial	New	^130,338^
Polymer to MMF	41° Ti-Cu mirror	1.05 1.89 1.69	/	/	/	/	/ (805) / (1310) / (1550)	6 × 6.5(W × H)	Serial	New	^220,221^
Polymer to SOI		**2.43**		**±** **2**	**30**	**±1.** **5**^b^	**±** **20 (1550)**				
SOI to SMF	Grating & lens	6.5	/	± 7.5	700	± 0.5	± 5 (1310)	275 (W)	Parallel	Custom	^207^
**Polymer to polymer**	**TPP mirrors**	**0.22 0.25**	**TE TM**	**±** **1.3**	**35**	/	**±** **200 (850)**	**10** **×** **30**	**Serial**	**New**	^140^
**Si**_**3**_**N**_**4**_ **to Si**_**3**_**N**_**4**_	**TPP mirrors**	**0.49**	**TE**	/	/	/	**390 (1300–1690)**	**140** **×** **80** **×** **24**	**Serial**	**New**	^139^
Si_3_N_4_ to SMF	TPP mirrors	1^a^	TE	± 2.2	20	2.2	300 (1340–1640)	140 × 80 × 24	Serial	New	^139^
Polymer to SOI	Imprint/GSL mirrors	3.6	/	**±** **2.5**	/	/	± 80 (1550)	320 × 14 × 19	Parallel	New	^58,59^
Laser to SMF	DNA pyramids	0.71	/	/	/	/	/ (450)	< 10 × 10	Parallel	New	^136^
Polymer to SOI	mirror & grating	18.7	/	/	/	/	/ (1265)	31 × 11 × 28	Serial	New	^131,339^
InP to SOI	TPP mirrors	2.5	TE	± 3	± 2.25	/	± 70 (1550)	82 × 38(W × H)	Serial	New	^142^
SOI to SMF	TPP mirrors	0.8 3	TE TM	± 2	19	/	> 180(1460–1640) > 130(1510–1640)	135 × 70 × 27	Serial	New	^141^

The bold values indicate that only simulation results were reported. The coupler found in ref. ^207^ was included because in Fig. 2b of that study, the concept is intended for a chip-to-board level coupling. Also, the longitudinal footprint from refs. ^58,139,141^ was reported to include the length of the inverse taper in the Si, Si_3_N_4_, and Si tapers, respectively

*BW* bandwidth, *L* lateral, *V* vertical, ∠ angular, / not reported

^a^Estimated based on a single mirror with μ-lens to SMF efficiency

^b^Angular dependence of the mirror angle

**Table 5:** Summary of SiPh inter-chip evanescent couplers in chronological order of publication date

Package	Taper Design	IL (dB)	Pol.	1-dB Toler. (μm)		1-dB BW (nm)	L × W (μm)	Thru.	Process	Ref.
				Lateral	Vertical					
SOI to a-Si^a^	Linear	0.2	TE	/	/	100 (1470–1570)	60 × 0.5	Parallel	Custom	^340^
**SOI to polymer**	**Nonlinear**	**0.4**	**TE**	**±** **3**	**/**	**±** **100 (1550)**	**200** **×** **5**	**Parallel**	**Custom**	^341^
**c-Si to a-Si**^a^	**Stacked ring resonators**	**< 0.2**	**TE**	**<** **±** **0.02 (bus-ring)**	**10.4(rise) 0.9(period)**	**<** **±** **5 (1555)**	**10** **×** **10**	**Parallel**	**Custom**	^342^
SOI to polymer	Nonlinear	**0.4**	**TE/TM**	**2**	**1.5**	100 (1470–1570)	1500 × 5.915	Parallel	Custom	^9,244^
SOI to a-Si^a^	Trident	0.49	/	/	**> 1**	30 (1530–1560)	295 × 0.9	Parallel	Custom	^343^
**SOI to Si**^a^	**Multilayer MMI**	**<1**	**TE**	/	**3.3(rise)**	**825 (1454–2259)**	**80** **×** **5**	**Parallel**	**Custom**	^344^
SOI to SOI^a^	MEMS	0.47	TE	/	0.2	120 (1460–1580)	30 × 1	Parallel	Custom	^345^
SiN to SiN	Linear	3.1	/	< ± 1	< 1	/ (1550)	500 × 3	Parallel	Custom	^163^
SOI to polymer	Segmented	1.25 0.5	TE TM	± 2	/	± 70 (1550) ± 60 (1310)	1500 × 6.5	Parallel	Custom	^155,346,347^
SOI to SiN^a^	Multistage	0.058	/	/	> 0.3	100 (1520–1620)	180 × 1	Parallel	Standard	^348^
SOI to SiN^a^	Linear	0.06	/	/	/	/ (1550)	23.6 × 0.85	Parallel	Standard	^349^
**SOI to polymer**	**Angled**	**0.2**	/	**>** **±** **5** **±** **1.5°(twist)**	**0.5**	**±00(1550)**	**200** **×** **15.3**	**Parallel**	**New**	^156^
**SOI to IOX**	**Segmented**	**<1**	**TE/TM**	**±** **4**	**3**	**±30(1550)**	**1500** **×** **12**	**Parallel**	**Custom**	^154^
SiN to IOX	Segmented	0.5	/	**>** **±** **4**	**<2**	75 (1515–1590)	2000 × 11	Parallel	Custom	^249^
Si_3_N_4_ to Si_3_N_4_	Linear	0.54	/	< ± 2	<0.8	400 (1200–1600)	1000 × 3	Parallel	Custom	^253^
Si_3_N_4_ to Si_3_N_4_	Segmented	0.36	TE	± 1.5	0.43(rise)	60 (1260–1320)	1500 × 0.71	Parallel	Custom	^56^
Si_3_N_4_ to polymer	Segmented	< 1	TE/TM	/	0.25(rise)	100 (1260–1360)	1000 × 4	Parallel	Custom	^350^
SOI to SiN_*x*_	Segmented	0.39	TE	± 1.56	1.1(rise)	160 (1480–1640)	520 × 1	Parallel	Custom	^153^

The bold values indicate that only simulation results were reported

*BW* bandwidth, / not reported

^a^Intra-chip coupling

**Table 6:** Summary of III-V intra-chip and inter-chip evanescent couplers in chronological order of publication date

Package	Taper design	IL (dB)	Pol.	1-dB Toler. (μm)		1-dB BW (nm)	L × W (μm)	Thru.	Process	Ref.
				Lateral	Vertical					
InGaAsP to InP^a^	Multistage	0.46	/	/	/	/ (1550)	310 × 9	Parallel	Standard	^351–353^
**InGaAsP to InP**^a^	**Segmented**	**0.18**	**TE**	/	/	/ **(1550)**	**570** **×** **3**	**Parallel**	**Standard**	^354–356^
**InGaAsP to InP**^a^	**Segmented**	**0.2**	**TE/TM**	/	/	/ **(1550)**	**40** **×** **3.4**	**Parallel**	**Standard**	^357^
InP to SOI^a^	Segmented	0.2	/	/	/	/ (1590)	180 × 1.7	Parallel	New	^358^
**AlGaInAs to SOI**^a^	**Multistage, resonant**	**0.02**	/	**>** **±** **0.2**	**>1**	**>** **± 100 (1550)**	**19** **×** **3**	**Parallel**	**New**	^359^
GaAs to SiN^a^	Linear	0.47	TE	/	/	400 (900–1300)	20 × 0.6	Parallel	New	^289^
**AlGaInAs to InP**^a^	**Segmented, resonant**	**0.18**	**TE**	**<** **±** **0.5**	/	**175 (1225–1400)**	**55** **×** **2**	**Parallel**	**Standard**	^360^
SOI to LiNbO_3_^a^	Linear	0.19	/	/	/	/ (1550)	150 × 1	Parallel	New	^50^
InGaAsP to SiN	Multistage	**0.32**	/	**± 1**	/	20 (1560–1580)	384 × 3	Parallel	Custom	^162^
AlGaInAs to SOI	Nonlinear	**0.3**	/	**±** **1.2** **±** **0.35°(twist)**	/	20 (1550–1570)	225 × 3	Parallel	Custom	^361^
**AlGaInAs to SOI**^a^	**Multistage, resonant**	**0.08**	/	/	/	**>** **± 50 (1550)**	**5** **×** **2**	**Parallel**	**New**	^362^
**AlGaInAs to SOI**	**Segmented**	**0.09**	/	**±** **1.1**	**>0.12**	**400 (1400–1800)**	**87** **×** **3.35**	**Parallel**	**Custom**	^363^

The bold values indicate that only simulation results were reported

*BW* bandwidth, / not reported

^a^Intra-chip coupling

**Table 7:** Summary of inter-chip cantilever couplers in chronological order of publication date

Package	Process	IL (dB)	Pol.	1-dB Toler. (μm)		1-dB BW (nm)	L × W (μm)	Thru.	Process	Ref.
				Lateral	Vertical					
SOI to SOI	Thermally induced strain	2.5 1.1	TE TM	± 0.625 ± 0.5	1.25	65 (1500–1565)	100 × 4	Parallel	Custom	^164^
SOI to SMF	Ion implantation	3.9 4.0	TE TM	/	/	160 (1450–1650) 170 (1480–1650)	9 × 13	Parallel	Custom	^165^
SiN to InP	Mechanical pressure	10.7	/	> ± 2	> 2	/ (1480–1600)^a^	750 × 10	Parallel	Custom	^173^
GaAs to SMF	Au/Ni/Cr layer induced strain	5.85 7.96 6.99 8.86	TE, 290K TE, 10K TM, 290K TM, 10K	/	/	75 (975–1050) 150 (900–1050) 75 (950–1025) 100 (875–975)	122 × 3.78	Parallel	Custom	^166^

All reported values in this table represent experimental results

*BW* bandwidth, / not reported

^a^Wavelength range where SOA gain was measured since a broadband source was used

**Table 8:** Summary of optical wirebonding or similarly categorized techniques

Package	Technology	Thru. (sec/link)	IL (dB)	1-dB BW (nm)	L × W × H (μm)	Process	Ref.
SOI to SOI	PWB	30–300	1.6	>300 (1280–1580)	2 (W)	New	^175–178^
Polymer to polymer	U-link	/	4.05 to 6.36^a^ 0.56 to 3.39^b^	/ (850)	(200–900) × (30–50)	New	^181–183^
SiN to SiN	3D ULI	400	>5.6^c^	/ (1550)	10 × 5 × 50,000	New	^163,193^
MMF to SMF	3D ULI	200	0.6	160 (1480–1640)	9 × 9 × 20,000	New	^364^
Paralyne C to SMF	Flexible paralyne	/	/	/ (450–680)	30 × 5 (W × H)	New	^184,185^
SOI to SOI	DOW	30	5.8	<10 (1590)	20 × 11 × 300	Custom	^179^
IOX to IOX	SmartPrint	/	6.08	/ (1310)	2000 × 3	Standard	^180^
Polymer to SMF	SCRIBE	75–244	0.45	> ± 100 (1550)	25 × 25	New	^64^

Throughput was calculated using the total write time reported for each connection. If total write time was not reported, then fabrication write speed, number of exposure passes, and connection length were used to calculate total write time

*BW* bandwidth, / not reported

^a^Evanescent coupler only

^b^Mirror coupler only

^c^Estimated based on SiN to ULI measured coupling loss

### Basics of optical coupling

**Fig. 7:**
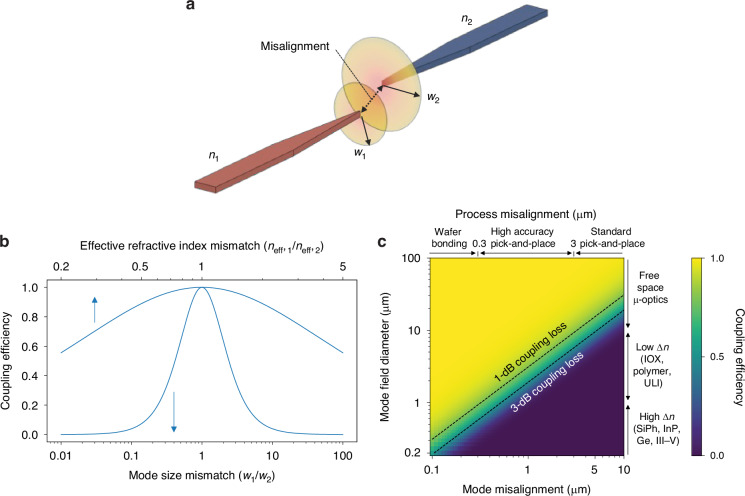
Plots showing the impact of critical parameters on coupling efficiency for a Gaussian incident and transmitted mode. In (**a**), a diagram showing the concept of modal overlap between an incident waveguide with *n*_1_ (*β*_1_, *w*_1_) and a transmit waveguide with *n*_2_ (*β*_2_, *w*_2_) where some misalignment exists between the two modes. Meanwhile in (**b**), the effect of mode size mismatch and reflections are highlighted while in (**c**) the effect of misalignment and mode field diameter size

The exact theory governing efficient coupling ultimately depends on the coupling design; however, a few common relationships can convey the relevant trends. To achieve efficient and alignment tolerant coupling, the incident and transmitted modes must be well size and shape matched, and reflections and misalignment should be kept to a minimum. The effect of these factors can be seen using the equation for coupling efficiency (*η*) between two overlapping modes^65^:

1 $$\eta =\left[\frac{4{\beta }_{1}{\beta }_{2}}{{({\beta }_{1}+{\beta }_{2})}^{2}}\right]\left[\frac{{\left(\int{E}_{2}{E}_{1}^{* }rdrd\phi \right)}^{2}}{\int{E}_{1}{E}_{1}^{* }rdrd\phi \int{E}_{2}{E}_{2}^{* }rdrd\phi }\right]$$

where *β*_1_, *E*_1_(*r*, *ϕ*) and *β*_2_, *E*_2_(*r*, *ϕ*) are the propagation constants (*β* = *n*_eff_*k*_0_ and *k*_0_ = 2*π*/*λ*) and electric field distributions for the incident and transmitted modes, respectively. This is shown schematically in Fig. 7a for incident and transmission waveguides with refractive indices of *n*_1_ and *n*_2_, respectively. While obtaining analytic solutions to this equation is challenging even for relatively simple scenarios, by assuming the incident and transmitted modes are roughly Gaussian ($E(r)={E}_{0}\exp (-{r}^{2}/{w}^{2})$ where *w* is the beam waist), we can numerically solve the integrals to gain insight. The results are shown in Fig. 7b which shows how mode size mismatch (*w*_1_/*w*_2_) and reflections (i.e., effective refractive index mismatch *n*_eff,1_/*n*_eff,2_) decrease coupling efficiency, with the size mismatch playing a more important role. Similarly, Fig. 7c highlights how modal misalignment impacts coupling efficiency while showing the typical scale of misalignments encountered for different fabrication processes such as wafer bonders, high accuracy pick-and-place tools, and ultra fast pick-and-place tools. Note that overlay misalignment in lithography is not plotted because the error is typically less than one-third of the critical dimension size (e.g., less than 30 nm for a 90 nm node process)^66^, and is thus can assumed to be less than 50 nm. It is interesting to note that symmetrically increasing the mode field diameter (MFD) of the incident and transmitted modes can increase alignment tolerance. As shown in the plot, the increase in MFD can occur by switching to material platforms which allow for a low refractive index contrast (Δ*n*) such as ion exchange (IOX) waveguides on glass or polymer core/cladding in an effort to increase evanescent field size. Switching away from high Δ*n* material platforms such as SiPh or InP has the downside of requiring larger waveguide pitches (to accommodate the larger MFD), and larger bending radii to maintain low loss routing across the die—both of which significantly impact the connection density of the circuit. This tradeoff—the desire for larger MFDs to increase alignment tolerance for chip assembly while simultaneously requiring smaller MFDs to increase connection and device density—is one of the most critical ones within photonic packaging. In the following sections, additional relationships critical to each coupling class will be presented in their individual sections, with the notion that modal overlap must be present in some level for every coupling type.

### Edge couplers

Edge or butt coupling involves aligning coupling elements on separate die along the direction of propagation such that light does not change planes vertically. Like the fiber-to-chip case, the couplers are typically located along the edge of the PIC and require processing such as deep reactive ion etching (DRIE), or dicing and polishing, to create high-quality facets^67^. In addition, for inter-chip coupling, a separate process is typically needed to etch a trench in the lower die to properly align the couplers in the vertical direction. The inverse process could also be used—selective deposition of a mesa on the lower die achieves the same goal. In either case, when well aligned it is the minimization of reflection at the chip interface and matching of the size and shape of the mode between chips which enables low coupling loss. Numerous strategies have been employed to achieve low coupling loss in the context of fiber-to-chip coupling, including tapers with linear and nonlinear profiles, multi-tip (trident) tapers, multilayer silicon nitride (SiN) assists, cascaded multistage tapers, 3D tapers, suspended tapers, and sub-wavelength gratings^68–74^. Similar techniques have been employed for the inter-chip case, with inverse tapers, multi-mode interferometers (MMI), and graded index (GRIN) lenses being the most promising.

#### Inverse and standard taper couplers

**Fig. 8:**
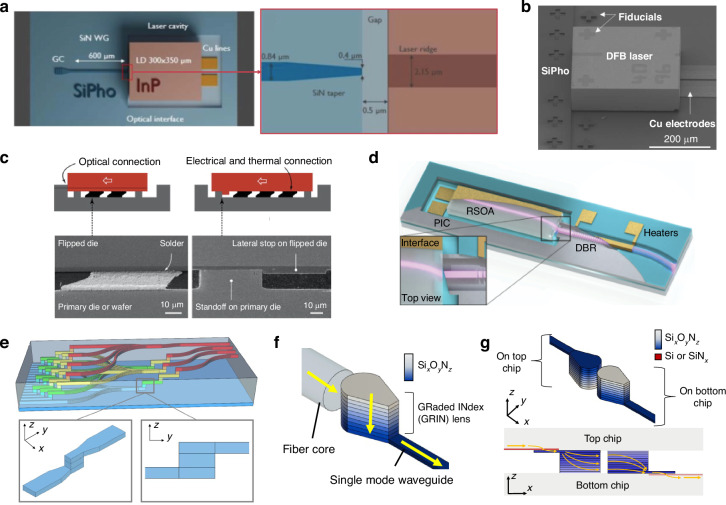
Examples of waveguide-to-waveguide couplers utilizing edge coupling. In (**a**) and (**b**), schematic and experimental images, respectively, of an inverse taper edge coupler from a SiN waveguide to an InP DFB laser passively assembled using pick-and-place tools (reprinted with permission from ref. ^75^ ©2023 IEEE). In (**c**), a side view of InP to SOI coupling is shown, where the red rectangle is the InP die and the gray etched shape is the SOI die. The black line across the InP and SOI is meant to show the waveguides on both surfaces being coupled. An experimental microscope image of the side view is shown on the bottom, demonstrating the submicron 3D alignment using mechanical stops (reprinted from ref. ^372^, ©2018 T. Barwicz et al. with permission from Elsevier and licensed under CC BY-NC-ND 4.0). In (**d**), a hybrid DBR laser based on flip-chip GaSb amplifiers and SOI waveguides is shown^48^ (©2022 N. Zia et al., licensed under CC BY 4.0). In (**e**), coupling between polymer waveguide layers using MMI devices^92^ (©2024 M. Weigel et al., licensed under CC BY 4.0). In (**f**, **g**), SOI to SOI or SiN_*x*_ to SiN_*x*_ flip-chip coupling using integrated GRIN lenses^322^ (©2025 D. Weninger et al., licensed under CC BY 4.0)

Inverse taper designs decrease the waveguide width at the interface between chips, thus expanding the MFD and enabling lower coupling losses and wider alignment tolerances. In ref. ^75^, an InP laser diode was pick and placed using passive alignment with ±0.3 μm accuracy onto a SiPh die (labeled SiPho in Fig. 8a, b), showing 1.5 dB insertion loss using a InP to SiN inverse taper. A similar hybrid integration example from refs. ^9,76^ demonstrated an InP PIC pick and placed onto a Si die and passively assembled using solder self-alignment with 3D mechanical stops as shown in Fig. 8c, expanding the effective lateral alignment tolerance to ±25 μm. Both nonlinear inverse tapers and metamaterial tapers were compared experimentally, demonstrating chip-to-chip losses as low as 0.25 dB and 0.9 dB for the inverse taper and metamaterial taper, respectively^76^. Coupler simulations^76^ showed a 1-dB vertical misalignment tolerance of only ±0.5 μm and measured solder self-alignment data showed that a deviation of ±0.5 μm between the die and interposer could cause zero self-alignment to occur^77^. Other inverse taper designs have used index controllable silicon oxynitride taper claddings to provide efficient mode transformation from an InP semiconductor optical amplifier (SOA) to a silicon-on-insulator (SOI) die^78^. Multi-tip inverse tapers have also been used to connect an InP die to an SOI PIC^79^.

Standard taper inter-chip edge couplers have also been used for chip-to-chip connections at mid-IR wavelengths^80–83^. For example, two die were mechanically aligned by interdigitating Cu nodules protruding from the chip’s edge and applying force horizontally to decrease the inter-chip gap size. To reduce index mismatch between germanium-on-silicon (GOS) waveguides, an arsenic trisulfide glass (*n* = 2.4 at *λ* = 8.4 μm) was dispensed at the interface^80,82^. Coupling losses of 4.1 dB at a 1.4 μm inter-chip spacing and 8.4 μm wavelength were demonstrated^81^. Couplers utilizing angled waveguides are also common when integrating active III-V devices to minimize back reflections into the gain area. Such a design is shown in Fig. 8d for a flip-chip, hybrid gallium antimonide (GaSb)-SOI laser at 2 μm wavelength with a simulated loss of 1.8 dB and estimated experimental loss of 5 dB^48^. Tapered spot size converters (i.e., standard tapers) can also be combined with the angled waveguides to improve coupling, such as in ref. ^84^, where an actively aligned GaSb-based SOA experimentally demonstrated 2.7 dB insertion loss to a SiPh die to form a hybrid laser from 1955 to 1992 nm wavelength. Simulated losses as low as 1.46 dB have been demonstrated for the same design and a similar wavelength regime near 1.9 μm^85^. Inter-chip edge coupling techniques have also been used in flip-chip hybrid integration of quantum cascade lasers (QCLs) with GOS waveguides for use in the 5.7–5.9 μm wavelength regime^86^ and *μ*-TP of GaSb-based gain elements to SiPh die for use near 2 μm wavelength^87^.

#### Multi-mode interferometer couplers

MMIs, traditionally used as on-chip splitters or combiners, can also be used to vertically couple between two separate layers of the same die or between two separate die. Such couplers, thus far only shown for intra-chip coupling, have included H-bar MMIs^88^, standard MMIs^89–93^, and edge assembled block MMIs^94^. The advantage of using MMIs is their inherent fabrication simplicity while allowing for vertical integration, at the expense of length sensitivity (due to the reliance on a mode resonance for efficient coupling) and longitudinal footprint. For instance, in refs. ^91,93^, and ref. ^88^, the total lengths of the MMI couplers are 3223 μm, 486 μm, and 241 μm, respectively. The vertical distance between waveguide layers can be significant though using MMIs, coupling over separations of greater than 20 μm with only 2.4 dB loss (which can be decreased to close to 1 dB according to simulation if lithographic alignment is improved)^93^. An image of a MMI design and its application to 3D photonic packaging are shown in Fig. 8e^92^.

#### Graded index couplers

Integrated GRIN lenses have also been proposed for efficient inter-chip coupling. Previously, SiON (also referred to as Si_*x*_O_*y*_N_*z*_) GRIN edge couplers were experimentally demonstrated with fiber-to-chip coupling losses <0.5 dB from 1520 to 1640 nm while using standard foundry processes^95^. An expansion of this design enables inter-chip connections with simulations showing <0.5 dB total loss with a 1-dB bandwidth of >360 nm. The proposed GRIN inter-chip coupler is shown in Fig. 8f, g, where GRIN lenses are fabricated on separate die and flip-chip bonded. Moreover, the inter-chip GRIN coupler does not require the coupling to occur at the edge facet, allowing for 2D arrays of inter-chip connections, and it is able to accommodate an inter-chip gap size of >10 μm with a wide underfill refractive index tolerance. The GRIN coupler can also be paired with evanescent couplers to provide a universal interface for heterogeneous integration and fiber-to-chip coupling across material platforms. These advantages come at the cost of increased fabrication complexity, especially involving film stress management across the wafer.

In summary, the use of edge couplers for inter-chip connectivity has a number of advantages. Edge couplers can achieve <1 dB of loss over a wide wavelength window with polarization independence, and foundries include low loss edge couplers as standard process design kit (PDK) components^96^. However, they have larger footprints along the propagation direction as adiabatic tapers or MMIs can extend >1 mm^9,76,78,88,91^, along with a low misalignment tolerance made worse by potential for substrate leakage. The requirement that they be located at the edge with a high-quality facet also precludes them from being used in 2D arrays, thus limiting their I/O density.

### Grating couplers

Grating couplers use a periodic etch with a period on the order of the wavelength of light to alter propagation direction by close to 90°. Parameters of grating couplers such as the grating period (Λ) and deflection angle (*θ*) are all directly tied to wavelength (*λ*) via the Bragg condition^97^:

2 $${n}_{{\rm{eff}}}-{n}_{{\rm{out}}}\sin \theta =\frac{\lambda }{\Lambda }$$

where *n*_eff_ is the effective index of the waveguide mode and *n*_out_ is the refractive index of the output medium. During fiber-to-chip coupling, the mode also typically undergoes spot size conversion to match the size of the waveguide mode, accomplished through an adiabatic taper. For inter-chip couplers, grating couplers are located on either die such that light enters into the lower die grating, diffracts upwards at an angle towards the upper die, and is coupled in plane upon incidence on the other grating. By changing optical propagation direction, the impact of the inter-chip gap size as well as its material (i.e., refractive index) are reduced. Enabling large vertical inter-chip gap sizes is important for several reasons: (1) it allows for simple electrical integration using standard Cu *μ*-pillar bumps or a similar variant, (2) it allows for thick BEOL claddings (>4–5 μm) with minimal effect on coupling efficiency, and (3) it increases the fabrication tolerance of the electrical interconnects (or other adhesive layer) as well as the BEOL thickness beyond the expected ±10%. The fact that grating couplers have been extensively studied in fiber-to-chip packaging^98,99^, are foundry compatible and found ubiquitously in PDKs^96^, and enable wafer level test, all combine to make grating couplers an appealing inter-chip coupling solution.

**Fig. 9:**
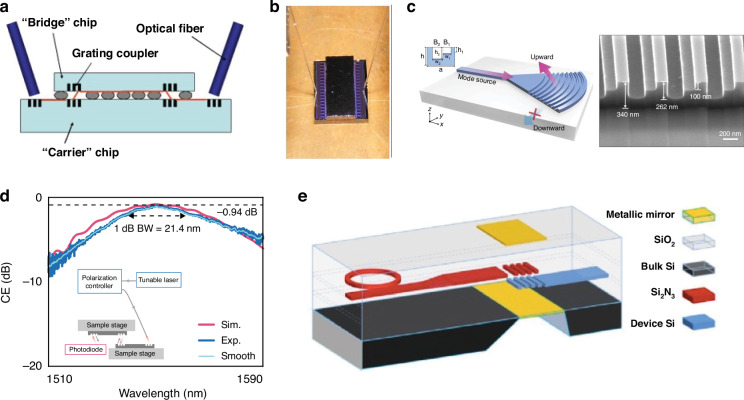
Examples of inter-chip grating couplers. **a**, **b** Grating-to-grating couplers using In bump self-alignment (reprinted with permission from ref. ^101^ ©Optical Society of America). **c**, **d** L-shaped grating for topological unidirectional resonance with inter-chip wavelength response^102^ (©2024 H. Wang et al., licensed under CC BY 4.0). In (**e**), SOI to Si_3_N_4_ intra-chip grating couplers using top and bottom reflectors are shown (reprinted with permission from ref. ^126^ ©Optical Society of America)

Several inter-chip grating coupler prototypes have been fabricated which highlight these advantages. Both Cu *μ*-pillar^100^ and In bump self-alignment^101^ have been used to achieve <1 μm lateral alignment of grating couplers on separate die. In ref. ^100^, the gap between the two die was 20 μm and was filled with air—demonstrating a high vertical and refractive index tolerance. In Fig. 9a, b, the cross sectional schematic and fully packaged system are shown for the In bump prototype^101^. A summary of inter-chip grating couplers is presented in Table 3. The presented data also illustrates a few of the challenges of grating couplers, including typical coupling losses above 3 dB, often made worse by substrate leakage for fully etched gratings. In addition, they suffer from high wavelength sensitivity with 1-dB bandwidths <±40 nm (with most <10 nm).

A variety of strategies are available for improving inter-chip grating couplers. As discussed in ref. ^102^, fiber-to-chip packaging designs have used L-shaped^103–108^, interleaved^109–111^, multilayered^112–114^, overlayed^115–117^, and tilt-etched^118,119^ strategies to minimize the wasted downward diffracted light. The use of a bottom mirror^120,121^, distributed Bragg reflector^122^, or bottom multilayer reflector^123^ have all shown lower coupling losses, limited substrate leakage, and wider bandwidth for fiber-to-chip couplers as well. The apodization of gratings, or changing the period along the propagation direction, also helps reduce back scattering and tailor the MFD and scattering angle^102^. Using an optimized L-shaped geometry with apodization, as shown in Fig. 9c, has shown results of 0.94 dB with a 21 nm 1-dB bandwidth near 1550 nm for inter-chip coupling, as shown in Fig. 9d^102^. This wavelength dependency may be improved by using high index guiding vias which have a simulated 1-dB bandwidth as wide as ±100 nm near 1550 nm^124,125^. Adding a bottom mirror, as shown in Fig. 9e, has also shown improved performance for intra-chip coupling^126,127^ and has simulated similar improvements for inter-chip coupling^128^. While high index vias or bottom mirrors are generally not desirable, strategies which can combine such reflectors in a foundry compatible fashion would allow grating couplers to achieve even wider appeal as complete inter-chip packaging solution.

### Free form couplers

**Fig. 10:**
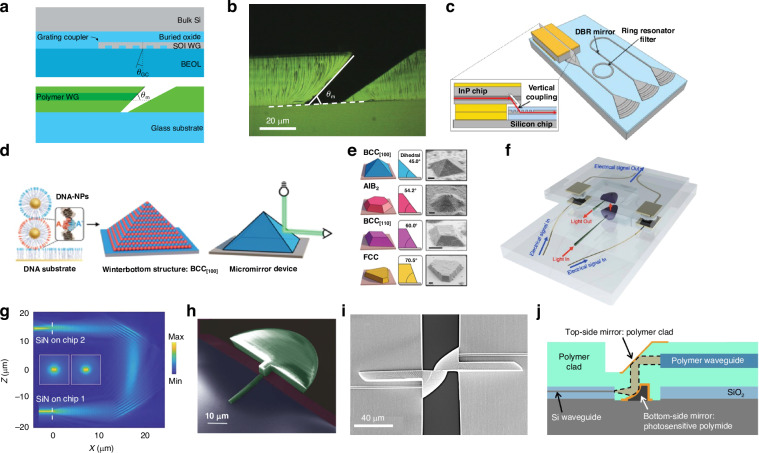
Examples of free form waveguide-to-waveguide couplers. In (**a**, **b**), a 45° TIR mirror in a polymer waveguide is used to couple to an SOI grating on chip (reprinted from ref. ^131^, with the permission of AIP Publishing). In (**c**), a combination of a TIR mirror in InP and an SOI grating couple light between pick-and-placed InP die (reprinted with permission from ref. ^335^ ©Optical Society of America). **d**, **e** DNA self-assembled pyramids (reprinted with permission from ref. ^136^, ©2023 American Chemical Society). In (**d**), a schematic of how DNA forms faceted structures which can be used as out of plane reflectors. In (**e**), SEM images show how the facet angles can be controlled during self-assembly. In (**f**, **g**), a schematic and simulation data for a flip-chip coupler using TPP fabricated micro-reflectors are shown^139^ (©2023 S. Yu et al., licensed under CC BY-NC-ND 4.0). In (**h**), an experimental image of the reflector is shown which was used for fiber-to-PIC coupling (adapted with permission from ref. ^141^ ©2024 Chinese Laser Press). In (**i**), an SEM image showing a TPP fabricated micro-reflector connecting two separate chips which are not flip-chipped, but placed side-by-side^142^ (©2024 H. Huang, licensed under CC BY 4.0). In (**j**), a side view of a coupler fabricated using greyscale and NIL is shown^58^ (©2022 Nakamura et al., licensed under CC BY 4.0)

Free form coupling describes the use of micro-lenses and mirrors to reflect light from an input waveguide out of plane and then reflect and/or focus the beam into the output waveguide on another die. Historically, semi-parallel fabrication techniques have been used for 45° mirrors, including laser ablation^129^ and dicing^130^ with specialized blades followed by a metal deposition to improve reflection. An example is shown in Fig. 10a, b where a 45° total internal reflection (TIR) mirror in an interposer polymer waveguide was formed using femtosecond laser ablation and the reflected light coupled to a chip level SiPh grating coupler^131^. Adding a microlens above the 45° mirror through RIE or polymer reflow can expand alignment tolerances, which is especially important for interposer to board connections where ball grid array solder bumps can have diameters of >100 μm^132^. Free form coupler designs using 45° TIR mirrors fabricated through other processes, such as angled focused ion beam (FIB), have been demonstrated as shown in Fig. 10c^133^. While FIB etching is often a serial process, it can be extended to parallel reactive ion etching (RIE) based processes with hard masks to create angled facets for vertical couplers^134,135^. Another approach is the use of self-assembled DNA pyramids as micro mirrors for out of plane coupling as shown in Fig. 10d, e^136^. The angle of the reflecting facet can be controlled by manipulating the lattice structure of the crystallite and the plane of that lattice structure which interfaces with the substrate^136^. Reflectivity can be further improved by coating the resultant self-assembled structures with a metal such as silver (Ag)^136^.

With the advent of multiple methods for 3D nanoscale lithography including TPP^23^, GSL, nanoimprint lithography (NIL)^137^, and thermal scanning probe lithography^138^, using curved micro mirrors and lenses for inter-chip coupling has become a feasible design strategy. An inter-chip coupler using TPP printed reflectors is shown in Fig. 10f, g^139^. Fabricating mirrors in RIE trenches is typical with free form couplers to center the reflector with the optical mode in the waveguide. In the simulations conducted in ref. ^140^, inter-chip coupling losses <0.25 dB were shown for coupling between polymer waveguides with a core (cladding) refractive index of 1.543 (1.525). Similar simulations in ref. ^139^ demonstrated <0.49 dB coupling loss between SiN waveguides. By using a curved reflector to simultaneously reflect and collimate, coupling losses can be significantly improved compared to 45° mirrors as wave front distortion is reduced^140^. These TPP based curved reflectors have been experimentally demonstrated for fiber-to-chip packaging as shown in Fig. 10h, coupling to a 0.220 × 1.5 μm SiN waveguide with a 4.9-μm-thick oxide cladding^139^ and to a 0.220 × 0.480 μm wide SOI waveguide with a 5.26 μm cladding^141^. They have also been experimentally demonstrated with <2.5 dB coupling loss for InP chip to SOI chip packaging without flip-chip bonding as shown in Fig. 10i^142^. Similar inter-chip couplers, shown in Fig. 10j, have been fabricated using GSL and NIL^58–62^. In this case, GSL was used to define micro-mirrors on an embedded SOI-PIC while NIL created 45° reflectors in a polymer RDL on an organic substrate.

One important benefit of employing mirrors and lenses is that they have low wavelength and polarization sensitivity. This can be seen by the 1-dB bandwidth and insertion loss columns in Table 4, showing bandwidths >±200 nm and operation from the visible (805 nm) and datacom/telecom regime (1310/1550 nm). Free form couplers also have small longitudinal footprints of <50 μm, although this is often larger in practice since adiabatic tapers in Si or SiN_*x*_ waveguides are needed to increase the MFD^59,139^. Another advantage is that the vertical alignment tolerance can be expanded to >35 μm allowing for ease of integration with electrical interconnects. The lateral alignment tolerance can similarly be expanded by increasing the mirror size and thus the MFD of the collimated beam. Of course, increasing the mirror diameter limits the lateral channel density, a tradeoff relevant for applications requiring connection densities >50 couplers/mm. While the thermal tolerance of such micro-optic elements needs to be carefully considered, as they are often fabricated from polymers with low glass transition temperatures, it is feasible to use resins which can withstand reflow temperatures of up to 250 °C^139^.

The primary drawback of free form couplers using TPP or GSL is their serial fabrication. Printing using TPP or GSL requires one field to be written after another, with current write times often on the order of several hours to write tens of couplers^23^. While parallel grayscale exposures are possible using chrome masks with computer generated bitmap data^143,144^, the photoresist development rate must be linearly proportional to the exposure dose, restricting photoresist selection to specialized, positive tone resists. For TPP, write time can be improved by over 3 orders of magnitude using techniques such as the shell-and-scaffold writing^145^, projection-based light sheet exposure^146,147^, and multiple-beam parallel writing^148–150^. While the outlook of these techniques is extremely promising, other parallel processes, such as using a TPP master mold for NIL or hot embossing, may be required to scale to high volume packaging involving >10^3^ optical connections.

### Evanescent couplers

Evanescent couplers transfer optical power between waveguides by bringing them in close proximity and having the modal distribution’s evanescent tails interact. A conventional directional coupler with two side by side, parallel waveguides is a well-understood example^97,151^. According to Saleh and Teich^97^, 100% power transfer is possible if zero phase mismatch between each channel is present and the coupler length is set equal to the specific distance at which 100% power transfer is predicted to occur. Modifying this design can allow for minimizing the transfer length and the allowable misalignment between waveguides, usually by using inverse tapers, similar to edge couplers. A single, linear inverse taper can be improved upon, for example, by using a segmented taper instead^152,153^. Segmented designs divide the taper into multiple linear sections where adiabatic transfer occurs only in the segment where the mode effective indices of the upper and lower tapers are well-matched. Nonlinear tapers achieve a similar result through a continuous profile, but fabrication can be more challenging due to the lithographic accuracy required. Segmented tapers have been used for inter-chip coupling between several different material interfaces such as SOI to SiN_*x*_-on-glass^152,153^, SOI to IOX waveguides-on-glass^154^, and SOI to a flexible polymer ribbon^155^. Results have shown coupling losses <0.5 dB with alignment tolerances >±1.5 μm.

**Fig. 11:**
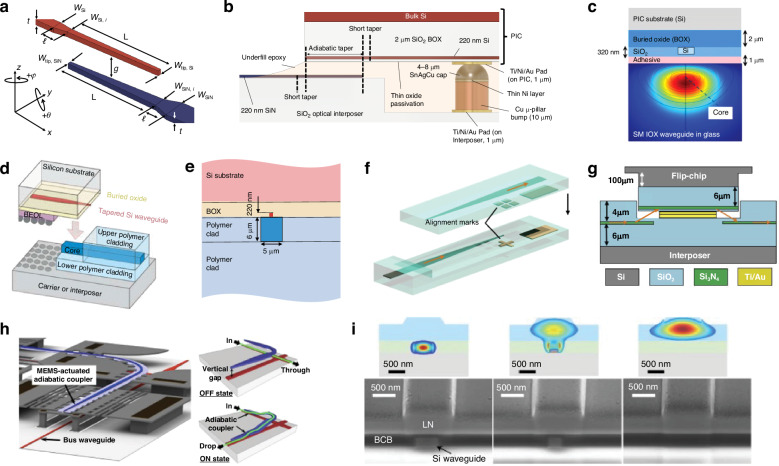
Examples of waveguide-to-waveguide evanescent couplers. **a**, **b** SiN on fused silica to SOI PIC (reprinted with permission from ref. ^152^, ©Optica Publishing Group). In (**a**), a perspective view of the coupler with the red indicating the double taper on the SOI PIC and the blue indicating the SiN double taper on the quartz interposer. In (**b**), a side view of an example fully packaged die showing the electronic-photonic integration of Cu micro pillar bumps as well as the use of an underfill epoxy for mechanical stability and refractive index matching. In (**c**), an IOX waveguide to SOI waveguide evanescent coupler cross section is shown along the propagation direction (reprinted with permission from ref. ^154^ ©2020 IEEE). **d**, **e** Polymer waveguide on board to SOI PIC. In (**d**), a perspective view of the coupler with the red indicating the multi-segmented taper on the SOI PIC and the blue indicating the polymer laminate on the board (reprinted with permission from ref. ^373^ ©2016 IEEE). In (**e**), a cross section of the evanescent coupler system with relevant parameters (reprinted with permission from ref. ^374^ ©2017 IEEE). In (**f**, **g**), SiN to SiN coupling with flip-chip Au–Au thermocompression bonding is shown including an oxide cladding opening on the interposer for decreasing the vertical gap between tapers (reprinted with permission from ref. ^253^ ©Optica Publishing Group). In (**h**), an SOI to Si evanescent coupler actuated by micro-electrical-mechanical system (MEMS) devices is shown along with how the coupler could be used as a switch for different layers or die (reprinted with permission from ref. ^345^ ©Optical Society of America). In (**i**), a cross sectional view of an evanescent coupler used to connect SOI waveguides to lithium niobate (LiNbO_3_) waveguides for modulator integration^50^ (©2019 M. He et al., reproduced with permission from SNCSC)

Other designs rely on angled tapers, where tapers on one die are intentionally rotated while tapers on the other are kept straight. As the die with the angled taper becomes translationally misaligned with respect to the straight waveguide, the angled taper remains overlapped with the same angle, thus not affecting coupling efficiency. Angled tapers expand translational alignment tolerance by at least 2.5 times compared to other couplers in Table 5, albeit at the expense of the pitch which is restricted to 15.3 μm for an angle of 4.4° and taper length of 200 μm^156^. A summary of different evanescent coupling designs can be found in Table 5, with a few of these shown in Fig. 11a–i.

In addition to taper design, the material systems used play an important role in determining footprint, sensitivity to cladding refractive index, and minimum pitch. For instance, in a high index contrast (HIC) system such as SOI, a segmented taper with a 500-μm-long adiabatic region is required to achieve approximately 3 μm alignment tolerances^152,153^. In a low index contrast (LIC) system such as IOX-on-glass, the segmented taper requires a 1.5–2 mm adiabatic region to achieve the same alignment tolerance^154^. Likewise, in a LIC system with a Δ*n* < 0.006^154,155^, the 1-dB tolerance for the adhesive refractive index was ±0.005 while for a HIC system with a Δ*n* ≈ 0.5–2^152,153^ this tolerance increased by 15 times. This sensitivity also applies to the thermal tolerance—in a LIC system, a 1-dB excess loss penalty can occur if the cladding, substrate, or adhesive index changes by as little as ±0.003 due to higher operating temperatures^154^. A LIC system also restricts the minimum pitch to be >20 μm while HIC systems can scale further to <10 μm. Even a conservative pitch of 10 μm for SiN_*x*_ or SOI waveguides results in a channel density of 100 couplers/mm compared to the 8 couplers/mm when fiber-to-chip packaging.

With that said, LIC systems remain desirable for low loss interfacing with SMF arrays, providing low propagation losses for optical redistribution, and fabrication maturity. For example, in ref. ^154^, the IOX-on-glass platform demonstrated an average of 0.68 dB insertion loss with 0.1 dB/cm propagation loss, while in ref. ^155^, the flexible polymer waveguides showed insertion losses of 0.3 dB (2.2 dB) at 1310 nm (850 nm) and propagation losses of 0.4 dB/cm (0.05 dB/cm) at 1310 nm (850 nm)^155,157^. Because these structures have also been fabricated and tested using foundry compatible materials, including reflow compatible laminates with siloxane based polymers^155^, and can be manufactured at the panel level, they are ideally suited for optical connectivity and redistribution at the board level following optical fan-out at the package level—a difficult challenge to address for future 3D photonic packaging technologies.

Furthermore, evanescent couplers have also been used for III-V-to-III-V or III-V-to-Si-PIC connections for integration of SOAs, lasers, or modulators. There exists a strong literature foundation in this area describing typical fabrication processes^158–161^, usually involving divinylsiloxane-bis-benzocyclobutene (DVS-BCB) wafer bonding of III-V to patterned Si followed by III-V patterning, or micro transfer printing of previously patterned devices using DVS-BCB bonding. Table 6 compares various evanescent coupler designs using wafer bonded or micro transfer printed devices, many of which use similar geometries to Si evanescent couplers. Vertical alignment tolerances are not typically reported, though the DVS-BCB layer thickness is usually <100 nm. The designs more common to III-V based devices are multistage couplers, which use multiple stacked layers for signal transfer, a technique useful when the refractive index difference between two waveguides is large or when large vertical gaps need to be overcome. For example, in ref. ^162^, a three stage coupler is designed to connect InP waveguides to Si_3_N_4_ waveguides which have an index difference of approximately 1.3–1.5. Multistage evanescent couplers were also used in ref. ^163^ to couple light between input Si_3_N_4_ interposer waveguides and SOI die waveguides, using a Si_3_N_4_ waveguide between the input and output layers. Such a multistage design could be used to overcome the thick BEOL layers by placing a taper structures midway through the BEOL as an intermediate layer.

In summary, as evidenced by the results in Tables 5 and 6, evanescent inter-chip couplers can achieve ultra low coupling losses of <0.5 dB across with bandwidths >100 nm and lateral channel densities >100 couplers/mm. These advantages can be had while using foundry compatible processes and, by optimizing taper design, can achieve micron scale alignment tolerances so automated pick-and-place assembly can be used. Evanescent couplers also have a few remaining challenges which future design improvements will need to address. For one, because they rely on the evanescent tail distribution, they are typically polarization dependent. In addition, while the longitudinal footprint can be minimized through taper design, these couplers still typically have longitudinal footprints of >100 μm due to the need for adiabaticity. Finally, while the vertical taper-to-taper gap can be effectively increased by using multistage taper designs, these still requires additional waveguide fabrication within the BEOL layers, adding to fabrication complexity.

### Cantilever couplers

**Fig. 12:**
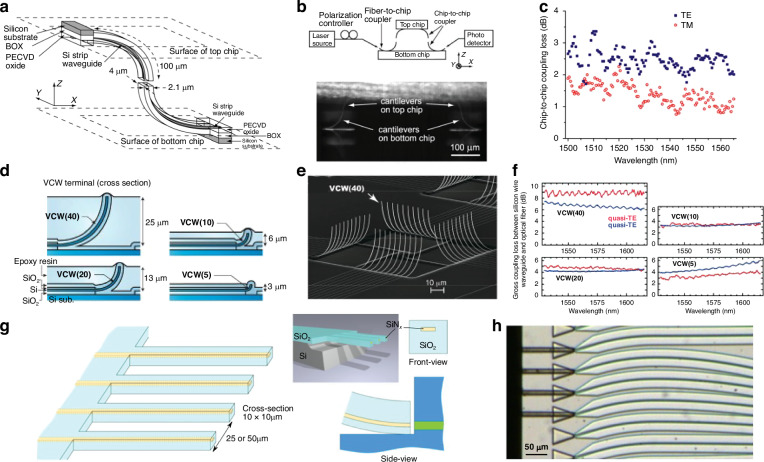
Examples of waveguide-to-waveguide couplers utilizing cantilever coupling. **a**–**c** Reference ^164^ (reprinted with permission from ref. ^164^ ©Optical Society of America). In (**a**), a schematic of the cantilever coupler. In (**b**), a side view of the packaged system showing the cantilevers on the top and bottom die and the measurement apparatus. In (**c**), the wavelength dependence of the cantilever coupler is shown for the TE and TM modes. **d**–**f** Reference ^168^ (reprinted with permission from ref. ^168^ ©Optical Society of America). In (**d**), a cross sectional view shows the material system of the ion implanted cantilever coupler for different cantilever taper lengths (VCW(40) stands for vertically curved waveguide with 40-μm long cantilever). In (**e**), a perspective view is shown of cantilever couplers fabricated using ion implantation. In (**f**), data shows the loss coupling from the cantilever to a lensed optical fiber for different polarizations and cantilever taper lengths (doubling these values can give a rough estimate for waveguide-to-waveguide coupling). In (**g**), schematics of a SiON cantilever edge coupler used for wafer level, automated probing of InP devices, and in (**h**), a top-down experimental image with SiON probe on the left and InP waveguides on the right, after passive assembly^173^ (©2021 X. Leijtens et al., licensed under CC BY 4.0)

Cantilever couplers are formed by inducing a tensile stress in a waveguide film and/or cladding that has been released or suspended, eventually leading to the waveguide bending in the out-of-plane direction in order to accommodate the stress buildup. The tensile stress can be induced by several processing techniques, such as a thermal treatment^164^, ion implantation^165^, or deposition of different films to increase stress^166^. When two PIC die are flip chip bonded and the bent waveguides are aligned, light can propagate as if it were traveling through an out-of-plane S-bend. An example of a cantilever inter-chip coupler utilizing a thermal treatment method is shown in Fig. 12a–c with associated experimental data^164^. In this study, SOI cantilevers were created by patterning, SiO_*x*_ cladding, and releasing the Si waveguides, and then annealing at 770^ ∘^C to achieve a 90° bend. The stress buildup occurs because of the stoichiometry—at high temperatures, the non-stoichiometric cladding releases impurities and densifies^167^, resulting in an increase in stress in the oxide cladding as compared to the 1 μm buried oxide (BOX) layer. Optical transmission data from Fig. 12c shows a wide 1-dB bandwidth from 1500 to 1560 nm. Although the vertical and lateral tolerances were only ±0.5 μm and 1.25 μm, respectively, these can be expanded by using an underfill adhesive with a refractive index closer to that of SiO_2_. Likewise, a standard 2–3 μm BOX could be instituted by depositing a thicker oxide cladding to increase stress buildup. Also, despite the high temperatures, data from ref. ^164^ of annealing temperature versus cladding thickness reduction suggests there is a temperature window from 200–400 °C which falls below BEOL thermal constraints, but is still hot enough to form cantilevers at <90° bends. This type of <90° cantilever was suggested as a method to improve post-bond vertical alignment in evanescent inter-chip couplers^156^, but further investigation is required in this area to verify its feasibility.

Ion implantation^165,168–172^ has also been used to induce stress and create cantilever couplers as shown in Fig. 12d–f. The fabrication process involved patterning, cladding, and releasing SOI waveguides, followed by ion implantation perpendicular to the cantilever surface. The cantilever cladding was removed during the release process while the remainder of the die stayed protected—a critical step since ion implantation of Si can dramatically increase the propagation loss (40 dB/mm for a dose of 2 × 10^15^ cm^−2^)^168^. The ion implanted cantilevers were also tapered from 430 nm at the waveguide to 190 nm at the tip over a lengths of 5–40 μm^165,168–171^. Once the tapers were bent, the entire system was then cladded with a 2 μm plasma enhanced chemical vapor deposition (PECVD) oxide and SiO_2_ index matched optical epoxy. A cross sectional view showing the material stack and fabricated couplers can be found in Fig. 12d, e. A minimum fiber-to-chip loss of ~3 dB was achieved for TE mode coupling from a lensed SMF at 1550 nm for a taper length of 5 μm (thus inter-chip loss would likely be ~6 dB). The data showing the effect of polarization, taper length, and wavelength can be found in Fig. 12f. While the loss is considerably higher than the thermally induced inter-chip coupler, the ion implantation coupler has an extremely compact footprint of <5 μm long and 3 μm high when bent.

The deposition of metal or dielectric layers along the surface of a suspended structure is also a method which can be used to for cantilever coupler formation. This concept has been demonstrated in suspended gallium arsenide (GaAs) waveguides with a polymer spot size converter being bent at 90° angles due to stress built up from Ni strips deposited on suspended GaAs^166^. To overcome the challenge of controlling the cantilever radii to better than ±10 μm, self-alignment structures involving hooks lead to a mechanical forcing of proper bend angle simply by changing the pattern mask. This mechanical structure preserved alignment for cryogenic temperatures when the film stresses would otherwise lead to further cantilever bending. The performance of such cantilever couplers when coupling to a 1.5 μm MFD SMF, can be found in Table 7 alongside other designs. While the measured insertion losses were high, measurements relied on light focused from an objective lens rather than direct fiber coupling, likely resulting in higher coupling loss. The vertical distance from the surface of the chip to the tip of the bent cantilever was also ~100 μm—a substantial vertical gap size. The measured insertion loss also changed by only ~2 dB when measuring at 10 K, indicating the usefulness of such a design during cryogenic operation.

The prototypes involving thermally induced strain, ion implantation, and metal induced strain show that fiber-to-chip cantilever couplers can be used inter-chip coupling by using flip-chip assembly. Cantilever based designs with a released waveguide on only one die have also proven useful for the development of novel probes for high speed photonic testing^173^. As highlighted by Fig. 12g, h, released SiN waveguides based on the LioniX TriPleX platform^174^ were used to couple to wafer level InP waveguides at a density of 40 connections per mm using self-aligning etched pits for passive assembly^173^. The throughput and robustness of the cantilevers are noteworthy, as an array of 32 cantilevers was able to be automatically aligned from a park position in less than 3 seconds and repeated over 2500 times without failure^173^. These couplers allow for large inter-chip gap sizes while using parallel fabrication processes and achieving low coupling loss. Additionally, if a large area were used to release a cantilever array, the minimum pitch would then be only limited by the cantilever MFD, allowing for high lateral channel densities of >100 couplers/mm. They do, however, include narrow lateral alignment tolerances, fragility in the absence of a sufficient cladding, and high temperature BEOL process requirements depending on the design used. To address these challenges, further studies are necessary. Technologies such as MEMS devices may offer higher control of bending radii or post-bond self-alignment, while different materials such as SiN_*x*_ might lead to wider alignment tolerances due to an increased MFD. This may also include the development of novel, stress controllable cladding materials to reduce thermal requirements and methods to limit the implicit tradeoff between ion implantation dose and optical propagation loss.

### Optical wirebonds

**Fig. 13:**
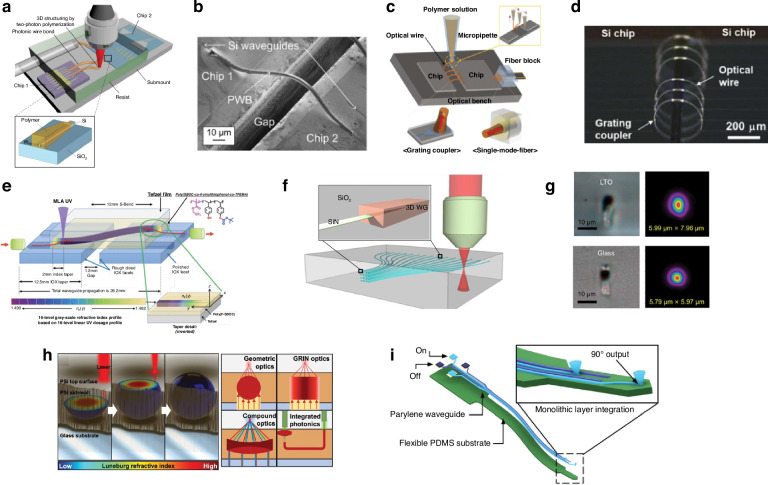
Examples of waveguide-to-waveguide coupling utilizing optical wirebonds. **a**, **b** Photonic wirebonds (PWBs)^178^ (reprinted with permission from ref. ^178^ ©Optical Society of America). In (**a**), the mechanism of TPP printing of PWBs for chip-to-chip coupling. In (**b**), an SEM image of one of the PWBs coupling SOI to SOI. **c**, **d** Direct optical wirebonds (DOWs) (reprinted with permission from ref. ^179^ ©Optica Publishing Group). In (**c**), the mechanism of printing the DOW for chip-to-chip coupling using a micro-machined glass nozzle. In (**d**), microscope images of the fabricated DOWs. In (**e**), a schematic view of SmartPrint technology including the graded index profile for the polymer waveguides and a cross sectional view of the IOX waveguides with polymer cross tapers (reprinted with permission from ref. ^180^ ©2022 IEEE). **f**, **g** 3D ULI waveguide couplers (reprinted with permission from ref. ^163^ ©2018 IEEE). In (**f**), a schematic of 3D ULI fabrication along with a zoomed in view of the end terminations for SiN-to-ULI waveguide coupling using adiabatic tapers. In (**g**), a cross section of ULI waveguides fabricated in commercially available eagle glass substrate and LPCVD oxide cladding with the measured optical mode profile. In (**h**), the SCRIBE technique is shown for forming free standing micro-optic devices in mesoporous silicon^64^ (©2020 Ocier et al., licensed under CC BY 4.0). In (**i**), a schematic of the flexible paralyne waveguide with mirror end facets for optical connections^185^ (©Reddy et al., licensed under CC BY 4.0)

Optical wirebonds are a versatile connector for low to intermediate volumes and are the optical equivalent to the electrical wirebond. These connectors can be polymer waveguides fabricated using TPP^175–178^, direct laser writing in mesoporous media^64^, polymer dispensing with open-to-air drying^179^, UV direct write lithography^180^, or flexible ribbon-based processes^181–185^. The first of these methods, TPP, produces couplers referred to as photonic wirebonds (PWBs), which can be found in Fig. 13a, b^175–178^. The chip-to-chip coupling performance showed a 1.6 ± 0.13 dB wavelength-independent coupling loss from 1530–1565 nm while the full spectrum from 1280–1580 nm demonstrated 2.5 ± 1.1 dB of loss. The primary advantage of PWBs is that because the structure is defined during the TPP process, which is completed after the two die have been bonded, the misalignment between waveguides becomes irrelevant. Robustness to mechanical vibration and shock was also demonstrated through a successful 1-meter drop test, which can be improved further by encapsulating PWBs in a rigid low index cladding.

The second method, the use of a dispensed polymer which dries when it comes into contact with air, has been termed the direct optical wirebond (DOW)^179^. In fabricating a DOW, a polystyrene powder dissolves in xylene solvent is dispensed from a glass nozzle. The speed and direction at which the nozzle retracted away from the surface determine the size and shape of the wirebond. As the polystyrene solution is dispensed and the nozzle pulled upward, the xylene rapidly evaporates and leaves a solid, optically transparent, well adhered, and geometrically tapered polystrene wire as shown in Fig. 13c, d. The best experimental insertion loss using this method was measured to be 5.8 dB at 1590 nm wavelength when using a wire terminated with a grating coupler. The 1-dB bandwidth was estimated to be less than 10 nm due to the resonance between input and output gratings. The 1-dB alignment tolerance of the DOW to the grating was demonstrated to be >2 μm in simulation. Unlike the PWB case, this was relevant because DOW is likely to be implemented using an automated dispense tool have tolerances of 1–10 μm. By rotating the nozzle 90° during the pulling motion, the DOW can be attached to orthogonal surfaces such as SMFs or chips attached in different orientations. One primary downside of a DOW is that, because of the nature of the deposition method, the control over the shape and size of the wirebond is greatly sacrificed when scaling to smaller feature sizes.

A hybrid between a PWB and an evanescent coupler is what is called “SmartPrint" technology^180^. This variant is made by bonding a Tefzel film to a glass substrate and patterning evanescent poly(F-SBOC) couplers (see ref. ^186^ for material information) using Heidelberg UV direct write lithography tools and spatially varying the exposure dose. This technique is useful for forming a 2D connection between different substrates as shown in Fig. 13e. The poly(F-SBOC) tapers are grayscale tapers, meaning the geometry remains rectangular but the refractive index is tapered from 1.482 to 1.490 by varying the exposure dose over a 2 mm length. As was discussed in “Cantilever couplers” section, because IOX-on-glass is a LIC system, precision control over the refractive index of the grayscale taper is required as the tip is designed to be 0.003 below the IOX waveguide core and the poly(F-SBOC) input is 0.01 above the IOX waveguide core. Still, the lateral misalignment tolerance is effectively irrelevant because alignment between poly(F-SBOC) tapers and IOX waveguides is done lithographically.

Another hybrid which is a combination of an optical wirebond and an edge coupler is a 3D ultrafast laser inscription (ULI) waveguide^187–191^. In 3D ULI, a waveguide is directly written in a transparent medium by scanning a pattern using a pulsed laser with a pulse width on the order of femtoseconds^192^. Because the pulses have an electric field strength which is roughly equal to the field strength binding valence electrons to the atom (order of 10^9^ Vm^−1^, or a laser intensity of 5 × 10^20^ W/m^2^), nonlinear absorption processes can cause avalanche ionization where electrons have enough kinetic energy to excite other electrons. This total energy is then transferred to the surrounding lattice to make permanent, local volumetric changes of the refractive index, creating waveguides^192^. Because the process relies on avalanche ionization, a wide range of transparent media can be used including PECVD or low pressure chemical vapor deposition (LPCVD) oxide claddings^163^. These waveguides have the benefit that they are instantly embedded in the material they are written within, and they can fabricated before or after die pick-and-place. Waveguides fabricated using 3D ULI have simulated interfacial losses to Si_3_N_4_ waveguides as low as 0.04 dB by terminating the ULI waveguide atop an adiabatic Si_3_N_4_ taper as shown in Fig. 13f, g^163,193^. Similar to other LIC systems, 3D ULI can form low loss connections to SMF arrays or MCFs and demonstrate propagation losses on the order of 0.3–0.8 dB/cm^163^.

Traditional 3D ULI can be taken further using the SCRIBE method^63,64,194^. This technique, shown in Fig. 13h, enables submicron control of the 3D refractive index profile by tailoring the amount of polymer remaining in a mesoporous silicon-based scaffold after exposure^63,64,194^. Steady improvements have resulted in SMF-to-SCRIBE waveguide losses of <0.45 dB with 3-dB alignment tolerances of >3 μm for SMF-to-chip vertical gaps of >37 μm^64^. The PWB, DOW, SmartPrint, ULI, and SCRIBE processes are highly customizable, making them well-suited for prototyping environments. However, their fabrication is still a serial process with connection densities <100 couplers/mm, limiting their current use to low volume manufacturing similar to electrical wirebonds.

Flexible polymer based ribbons also offer a way to make connections across large distances with a pre-patterned component. The flexible ribbons usually terminate with a type of coupler mentioned elsewhere in “Types of waveguide to waveguide couplers” section such as a free form or evanescent coupler. For example, in refs. ^181–183^, die-to-board connections were made through a board level evanescent coupler and a die level 45° TIR mirror fabricated via dicing with a double 45° V shaped blade. The evanescent coupler was created by removing the waveguide cladding on the ribbon and board, and pressing the ribbon into the board waveguide with a tool tip of a specific radius, resulting in coupling ratios of approximately 0.3–0.65 (6.36–4.04 dB loss). Another flexible ribbon includes that found in Fig. 13i which uses paralyne C waveguides with 45° reflectors at the waveguide termination^184,185^. This design also allows for electrical integration on the ribbon by depositing platinum traces atop a PDMS cladding. Simulations of the 30 lowest order modes for a paralyne C waveguide showed <3 × 10^−10^ dB/cm additional losses caused by the platinum layer interactions for the fundamental mode. This type of opto-electronic flexible ribbon could be used for implantable or wearable biophotonic devices used to probe tissue which otherwise would cause damage when integrated using rigid substrates^195^.

## Photonic vias

Thus far, the discussion has been in the context of inter-chip couplers which transfer light from the surface of one die to the surface of a separate die; however, another category of couplers are those which connect waveguides on the front and back surface of the same die by transferring light through the substrate. This also includes couplers which connect waveguides on separate die by guiding light through a third substrate in between them. These photonic vias are distinct from simple intra-chip couplers, as intra-chip coupling distances are typically short (i.e., <5 μm) and can be accomplished through typical evanescent^196^, grating^126^, or edge coupling^88^ techniques. We can further categorize photonic vias into two different types: guided mode vias and free form vias. In guided mode vias, a via is etched and filled to form a vertical waveguide which light is then coupled into. In a free form via, light is reflected and collimated using micro-mirrors, but is not guided by a waveguide inside the via. In the next two sections, designs for each type of coupler will be presented.

### Guided mode vias

**Fig. 14:**
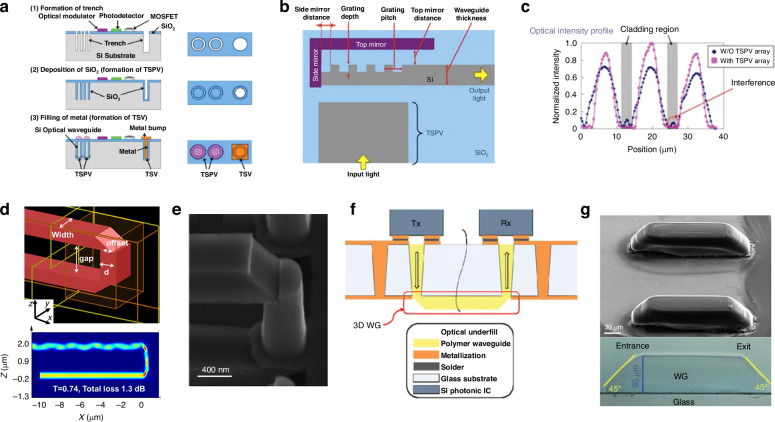
Examples of guided mode photonic vias. **a**–**c** Si guided mode vias using a grating coupler (reprinted with permission from ref. ^197^ ©2011 IEEE). **d**, **e** A Si guided mode via using a 45° reflector (reprinted with permission from ref. ^163^ ©2018 IEEE). **f**, **g** A polymer guided mode via using 45° reflectors (reprinted with permission from ref. ^201^ ©2015 IEEE)

To date, there have been few examples of guided mode vias explored. Those that have been developed can be further subdivided into polymer based vias and silicon based vias. A potential fabrication process for a silicon based via in thin Si substrates was outlined in ref. ^197^ and is shown in Fig. 14a. This process is executed during the same step as the etching of the electrical TSVs, where a DRIE step with an SiO_2_ hard mask simultaneously etches electrical TSVs and through silicon photonic vias (TPSVs). The diameter of the Si core of the TSPVs are 5 μm and the etch depth is 50 μm. The TPSV trench was filled with SiO_2_ to isolate the Si core and form a vertical multimode Si waveguide. Similar to a typical electrical TSV process, the backside of the wafer is thinned such that the bottom of the 50 μm TPSV is exposed. A cross sectional image showing the TSV and TSPV alongside one another can be found in Fig. 14b. This process results in the measured near field pattern (NFP) is shown in Fig. 14c, which demonstrated high confinement of light inside the Si core region versus the die which had no TSPV at all. To couple light into the TSPV at the entry or exit, the use of Si grating couplers with Ag reflectors was proposed. Simulation results using 2D finite-difference time-domain (FDTD) show coupling efficiencies as high as 73.7% (1.32 dB loss) for TE polarized 1550 nm light when using the Ag reflector, and efficiencies as low as < 5.5% (11.3 dB loss) when no mirror or TSPV were used. These efficiencies were for one end of the via, so additional loss is expected when including both input and output. Based on the reported data, the TSPVs aid in modal confinement, but are not necessary to obtain moderate coupling efficiencies (>50%). This is important when considering the process constraints required for DRIE as well as wafer thinning procedures.

Another silicon guided mode via design uses 45° SOI TIR reflectors to couple light in and out of a-Si vias formed in SiO_2_ claddings as shown in Fig. 14d^163^. By optimizing the mirror offset (75 nm), the gap (1.3 μm), and the via width (*d* = 450 nm in Fig. 14d) losses as low as 1.3 dB were achieved^163^. The fabrication involved tetramethylammonium hydroxide (TMAH)-based Si etching to form 45° reflectors, followed by RIE and a-Si deposition to form the via^198^—fabricated vias with reflectors can be found in Fig. 14e. Although the via was fabricated in an SiO_2_ cladding, a similar concept could be used in thin glass substrates. The challenge for via formation in thin glass would be the high aspect ratio deep SiO_2_ etching (>50 μm) required to form single mode a-Si waveguides with diameters <1 μm. Additional studies would also be needed to ascertain the via propagation loss as simulations in ref. ^163^ suggest the vias are lossy (>3 dB) above 2-μm thicknesses. Lastly, with the advent of TPP, GSL, and NIL, implementing more efficient reflectors could provide improvements in coupling efficiency and bandwidth.

Polymer based vias offer another option for coupling of light through a chip^199–202^. Polymer via designs also use 45° TIR mirrors used to couple light into the via as outlined in Fig. 14f. An example fabrication process outlined in ref. ^201^ utilized thin glass substrates (150-μm thick), with inclined lithography to pattern the 45° TIR mirrors, resulting in the mirrors shown in Fig. 14g. Inclined lithography offers advantages over GSL and the moving mask method because it does not require a precise exposure gradient and positive and negative photoresists can be used. Furthermore, 2D FDTD simulations determined the losses at 850 nm for a 60 μm diameter via (*n* = 1.511) in a 100 μm glass substrate (*n* = 1.503) capped with 50 μm square multimode polymer input and output waveguides. The data showed that 0.5 dB loss was achievable if the mirror angle was kept within ±5° of 45°. Experimental data from ref. ^202^ demonstrated the vias also show <1 dB of loss for thicknesses >100 μm as well. Fabrication challenges of this design included difficulty in patterning 45° features in air due to the large index contrast, a zero gap mask requirement, and multiple exposures. Solutions include partial substrate immersion in water during exposure and using the glass substrate simultaneously as the mask by having a deposited Cu layer act as a zero gap mask. These solutions provide initial steps towards a parallel lithography technique for angled features, although the process has thus far been constrained to thin with non-transparent substrates being an open challenge. Further study is also needed to address increasing the polymer refractive index and reducing via diameter, leading to higher channel densities. Development of guided mode vias with LIC foundry compatible materials such as SiO_2_, SiON, or SiN will also enhance integration and adoption. This may require innovative thin film deposition techniques to eliminate voids or high index spin-on variants, like spin-on-glass^203^ or polymer via filling^204^.

### Free form vias

Designs for free form vias typically involve a TPP or GSL mirror, a 45° mirror, or a grating to reflect light at 90° into the substrate. From there, the opposite surface may have another reflector for coupling between layers of the same chip, or it may have a micro lens to collimate the beam such that it can be directed towards a third, flip-chip bonded die. Designs tackling the latter challenge are important for future 3D integrated photonic packaging, because much of the current focus hasted rested on chip-to-chip or chip-to-interposer coupling without addressing optical chip-to-board and interposer-to-board connections. Two factors make this difficult: a sizeable vertical distance (>>100 μm) and a large refractive index difference between on-chip or on-interposer waveguides and board level waveguides. Designs utilizing free form vias could provide a path forward to overcoming these two challenges in future optical printed circuit boards using fiberless technology. Moreover, a variety of studies have investigated the performance of fully etched air vias versus propagation through transparent substrates^205–208^. Because different interposer materials are all widely used including silicon, glass, and organic substrates, separate sections will cover each situation individually.

#### Glass interposer vias

Glass interposers are appealing due to their advantageous thermal, mechanical, electrical, and optical properties. In addition, glass can be processed at the panel level, providing a scalable option in terms of cost and throughput. For free form vias, glass interposers have potential due to their high transparency in the visible and NIR wavelength regimes. This helps to eliminate the design and fabrication challenges associated with determining a suitable optical via material and etching through glass vias (TGVs). To elaborate, a number of methods have been explored to etch high quality TGVs including sand-blasting^209^, photosensitive glasses^210^, focused electrical discharging^211^, DRIE^212^, laser ablation^213^, and deep wet etching^214^. Of the most promising are wet etching processes whose directionality is enhanced by pulsed laser exposure. These techniques involve either inducing microstructural changes followed by wet etching^215^ or locally exposing an absorbing organic solution such that the glass is melted at the solution interface^216^. The drawback of such techniques is that they are serial processes compared to the Bosch process utilized in TSVs. Parallel processes are available, such as UV exposure and baking of photosensitive glass substrates, which changes the exposed area to a ceramic to be wet etched and form high aspect ratio features^217^. The extinction coefficient *k*, and therefore propagation loss, has not been characterized for HIC systems on photosensitive glass though. Therefore, optical TGVs which do not require such etching is an advantage and helps make the design agnostic to the type of glass used.

**Fig. 15:**
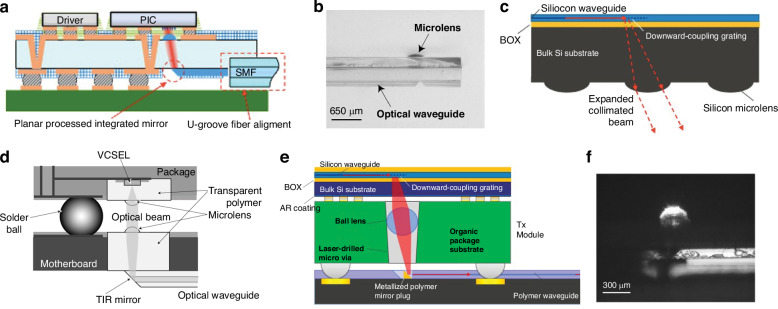
Example of free form photonic vias. In (**a**), a photonic via for coupling through a glass interposer using a back side TIR mirror with a top side lens and on-chip grating coupler (reprinted with permission from ref. ^208^ ©2016 IEEE). **b**–**d** OptoBump technology for coupling through an through a quartz substrate (**b**) using diced 45° TIR mirrors and dispensed microlenses (reprinted with permission from ref. ^218^ ©2003 IEEE). In (**c**), a surface grating with a backside etched Si microlens is used for through silicon optical coupling (reprinted with permission from ref. ^207^ ©2019 IEEE). In (**d**), the same OptoBump technology shown in (**b**), except for an organic substrate (reprinted with permission from ref. ^218^ ©2003 IEEE). **e**, **f** A grating coupler, ruby-doped sapphire ball lens inserted into a cavity formed by laser ablation, and a metal-coated polymer mirror were used for coupling through an organic substrate (reprinted with permission from ref. ^206^ ©2020 IEEE)

A common design strategy for free form glass vias is to have a 45° mirror on one side of the die and a microlens on the opposite side for collimation and focusing^208,218,219^. The design found in Fig. 15a^208^ shows this strategy being used for coupling with simulated losses below 2.43 dB from an interposer polymer waveguide to an SOI PIC waveguide^220^. The 45° mirror was fabricated using inclined lithography and a 60 μm diameter polymer plano-convex was formed using polymer reflow at 150 °C^221^. Yet another design with simulated coupling losses <1 dB through a 550-μm-thick quartz substrate can be found in Fig. 15b^218,222^. Here, the 45° TIR mirrors were formed through 90° V-shaped diamond blade dicing of epoxy waveguides and the 352 μm diameter microlenses were formed by UV epoxy dispensing. This type of strategy has also been used for coupling IOX-on-glass interposer waveguides to an SOI PIC^219^. The 45° mirrors in this case were fabricated using laser ablation^223^ while graded index IOX, CO_2_ laser melting, and RIE of reflowed photoresist masks were explored for micro lenses creation^219^. In a mirror and microlens design, critical process requirements include the TIR mirror angle, the lens to mirror alignment, and the PIC to interposer alignment. For example, in ref. ^208^, the TIR mirror angle needed to be 45 ± 4 degrees for mirror to lens loss of <1 dB and the lens to mirror alignment needed to be <3 μm for similar loss.

The presented designs represent viable options for coupling light through glass interposers without needing to form guided mode vias. However, these designs only illustrate transfer of light through glass—to the authors’ knowledge there is not a free form design demonstrating coupling between integrated waveguides in the same die. Further investigation on using reflecting micro mirrors patterned using TPP or NIL, or multistage evanescent couplers, may provide different avenues for reaching higher performance and levels of integration.

#### Silicon interposer vias

Another appealing material for package substrates is silicon, which offers foundry compatibility with the possibility of high density electrical I/O through TSV technology. The primary drawback of Si interposers are their high cost, currently limiting their availability to applications outside of high performance applications. In terms of free form Si vias, the design employed in ref. ^205^ used a 45° board level mirror to reflect light through an Si interposer to an on-chip curved micro-mirror described in “Free form couplers” section. Simulations determined the MFD incident on the curved micro mirror after traversing a 200 μm Si interposr to be approximately 14 μm. Based on this, a maximum coupling efficiency of 84% was simulated for transmission from the PIC to the board. The NFP and far field patterns (FFP) were experimentally measured, and the mirror element was observed both collimating (24.6° by 30.2° FFP without mirror versus 10.7° by 10.1° FFP with mirror) and expanding the MFD (3.9 μm by 3.6 μm NFP without mirror versus 5.8 μm by 6.3 μm NFP with mirror).

A separate design utilizing an on-chip grating coupler with a bottom reflector and backside etched Si lens to focus the beam onto a board level mirror was experimentally tested in the O-band and C-band^207,224^. This design is shown schematically in Fig. 15c. The micro-lenses were formed by patterning and reflowing photoresist followed by RIE. The optical power coupled from an angled-polished SMF was compared to a reference system composed of coupling through a thinned 100 μm Si substrate with a standard grating coupler. An additional 3 dB coupling loss was observed where 1.85 dB was attributed to Fresnel loss and 1.15 dB to the microlens interface. The 1-dB alignment tolerance from fiber-to-chip was as follows: ±2.5 μm lateral grating to microlens tolerance, ±7 μm lateral, ± 0.5° angular, and 700 μm vertical microlens to fiber tolerance.

#### Organic interposer vias

Organic materials offer an even lower cost alternative to glass or silicon interposers at the expense of density and performance^225^. Most of the organic interposer vias referenced here involve removing a section of the interposer by a dicing or milling process to allow for optical transmission. Similar to the section “Glass interposer vias”, many designs utilize either a TIR or metal-coated board level mirror with a microlens to provide increased alignment tolerance from interposer to board^206,226–232^. However, many demonstrated designs for organic substrates terminate at the die level with a vertical cavity surface emitting laser (VCSEL) or vertical photodetector instead of coupling to an on-chip waveguide. Such a design for a polymer interposer is shown schematically in Fig. 15d.

One exception was in ref. ^206^ which utilized a die level SOI grating coupler to form chip-to-board connections as shown in Fig. 15e, f. A 400-μm-thick glass fiber reinforced organic package substrate was used and a via was created by laser ablation with measured taper angles of 1.6° ± 0.5°. A ruby-doped sapphire ball lens with a diameter of 300 μm was placed in the via and active alignment of interposer-to-board and chip-to-interposer was used for assembly. The measured chip-to-board loss was approximately 3.4 dB including grating coupler, ball lens, and board reflector with a 1-dB lateral alignment tolerance of ±7 μm. Further improvement of this chip to board loss by 1.7 dB was shown to be possible by improving the directionality of the grating coupler^233^, and implementation of other reflective elements outlined in “Types of waveguide to waveguide couplers” section could provide a path forward for wider bandwidth connections.

## Applications

The introduction of the aforementioned couplers from “Types of waveguide to waveguide couplers” section and vias from the section “Photonic vias” to optical systems presents an opportunity to accomplish several objectives. First, these couplers enable optical fan-out. This is the idea of decreasing the I/O pitch at the die level by introducing waveguides and couplers at the package level whose job is only to route the signal from the die to SMF arrays. Because optical fan-out enables orders of magnitude difference between the coupler pitch on the die and at the SMF array while maintaining low propagation loss and low fiber-to-package coupling loss, it allows for the mass parallelization of optical connections. The ability to scale the number of parallel optical connections is a vector currently unavailable to integrated photonic transceivers. Thus, these couplers are increasingly relevant for scaling data capacity in tele- and data-communications networks using state-of-the-art co-packaged optics (CPO) transceiver architectures—and in turn are critical to the artificial intelligence models relying on such cloud infrastructure. Therefore, the implication of using such couplers in CPO systems will be discussed in the section “Datacom and telecom co-packaged optics switch packages”.

The second, but equally as important, the couplers offer the possibility of “fiberless” system level optical connectivity between photonic sources, modulators, switches, processors, amplifiers, and detectors, of which some or all could be on different, optimized material platforms. While *μ*TP or hybrid, heterogeneous, or monolithic integration all consider combining of different material platforms, they are often focused on doing so at the device or die level. However, the inter-chip couplers and photonic vias presented here provide an opportunity to not only accomplish this material integration at the device or die level, but also at the system level. In fact, they enable a second capability on top of system level optical connectivity—they make it possible to have a pluggable or detachable optical chiplet. As will be described in the section “Biochemical sensing”, such interfaces are especially useful in PIC based biochemical sensing where it is desirable to have a system where low cost PICs are disposed of without simultaneously disposing the expensive sources or detectors. All in all, this type of enhanced connectivity is critical to many current photonic applications such as photonic neuromorphic computing, optically connected memory, integrated quantum photonics, and LiDAR systems; thus, the presented couplers will be discussed in the context of such systems in the sections “Photonic neuromorphic computing”, “Optically connected memory”, “Integrated quantum photonics”, and “Heterogeneous integration of components for compact LiDAR systems”, respectively.

### Datacom and telecom co-packaged optics switch packages

By 2025, the global datasphere is estimated to grow to 175 Zettabytes, with 46% of the world’s stored data residing in public cloud environments^234^. To meet demand, data center top of rack switch packages have doubled total bandwidth capacity every two years, with 51.2 Tbps switch packages using 512 electrical I/O, each operating at 106.25 Gbps per lane using the IEEE 802.3-ck standard, being commercially available^235,236^. Further standardization of 200 Gbps per lane is already underway^237,238^. However, the power consumed by centimeter long board level Cu traces and the limited pluggable transceiver pitch make continued scaling difficult^239,240^. This is critical when considering the energy consumption of data centers. For example, data centers already accounted for 18% of all energy consumption in Ireland in 2022 and by 2031 roughly 15% of Denmark’s energy consumption will be due to data center power usage^241^.

**Fig. 16:**
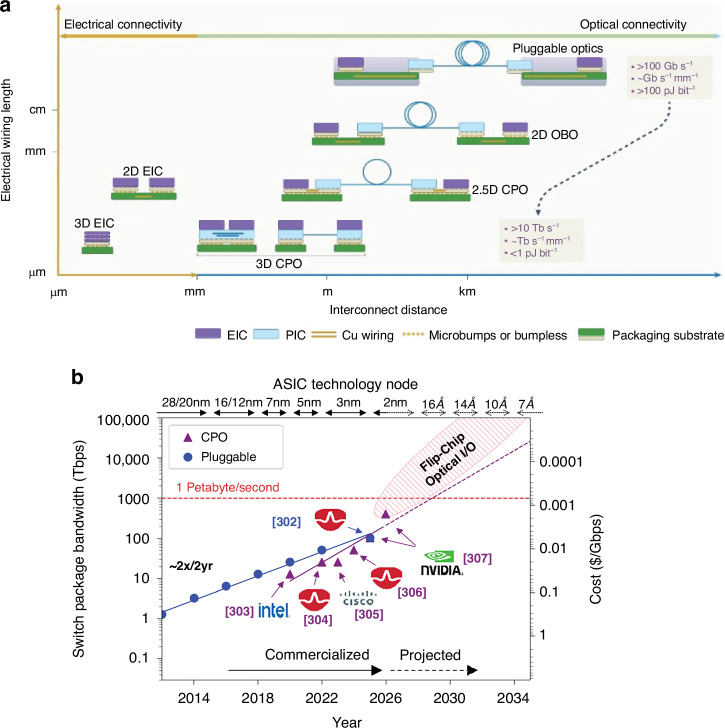
Scaling trends for bandwidth, cost, and energy efficiency in datacom and telecom interconnects. In (**a**), a plot showing different switch system architectures with varying levels of electronic-photonic integration at the package level ranging from pluggable optics to 3D CPO^242^ (reproduced with permission from SNCSC). In (**b**), a plot representing the change in scaling caused by the introduction of integrated, flip-chip optical I/O. The solid blue line shows the exponential increase in commercial switch package bandwidth which has occurred over the past decade due to pluggable transceiver systems^235,375,376^. The dotted purple line shows projections over next decade using the scaling trend from CPO. The right axis signifies this improvement in package bandwidth has been accompanied by an improvement in the cost per bit (per second). The top axis signifies that each successive improvement in switch package bandwidth also requires a more advanced technology node to scale ASIC performance. The callouts highlight the manufacturers who have successfully commercialized CPO systems^377–381^. The hatched pink circle demonstrates how the adoption of flip-chip optical I/O not only continues exponential scaling, but provides an increase in scaling rate which can dramatically accelerate the development of switches with >1 Pbps package I/O bandwidth

The need to reduce Cu length has forced a transition to CPO where the SiPh transceiver is on the same package substrate as the application-specific integrated circuit (ASIC), as shown in Fig. 16a^242^. Scaling using this architecture has its own challenges, including the serial active alignment and bonding of SMF arrays to PICs. The assembly challenge is further compounded by the potential need for >10^3^ SMFs to scale to Pbps bandwidth capacities by 2035 as required by current trends^235^. For example, a 1.6 Pbps switch package containing a central ASIC surrounded by 16 transceivers would require 4000 SMFs assuming 200 Gbps per wavelength with 4 *λ*/channel. These volumes drastically increase if lower lane data rates from 25 to 100 Gbps per wavelength are used. Considering the limited SMF array channel density of 8 connections/mm, a maximum of 320 fibers could be connected to a 10 mm × 10 mm PIC.

**Fig. 17:**
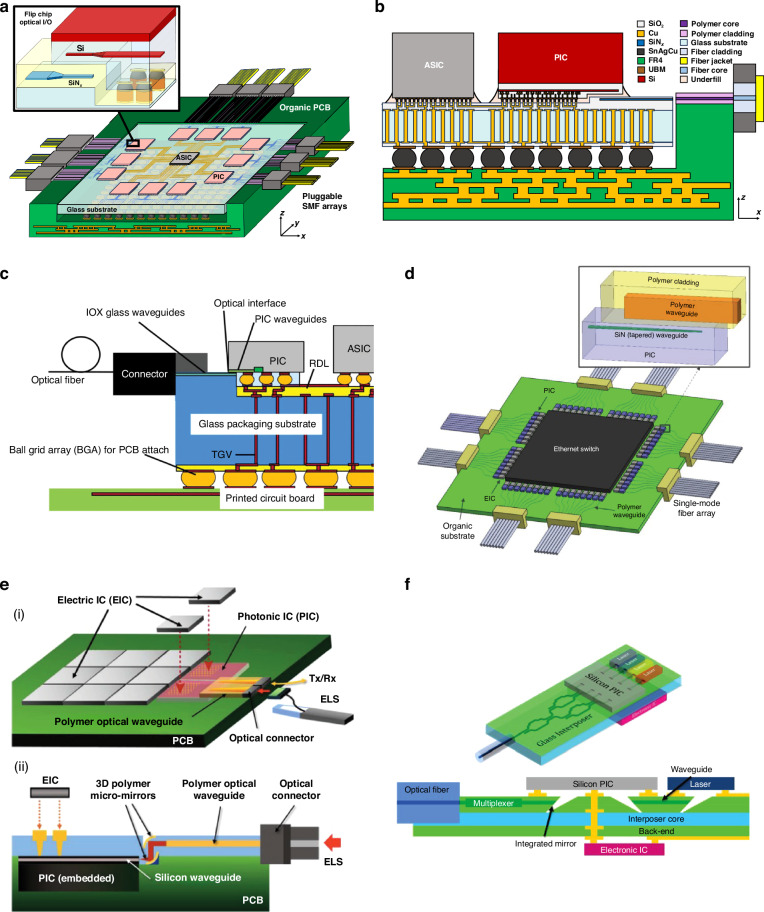
Examples of inter-chip couplers being implemented to improve co-packaged optics switch package performance. In (**a**) and (**b**), flip-chip SiN_*x*_ to SOI evanescent couplers with Cu *μ*-pillar bumps for Pbps I/O^153^ (©2025 Weninger et al., licensed under CC BY 4.0). In (**c**), IOX to SOI evanescent couplers integrated with solder bumps and TGVs for optical and electrical fan-out (reprinted with permission from ref. ^249^ ©2023 IEEE). In (**d**), an organic package substrate contains polymer waveguides connected to SiN waveguides on transceiver PICs using a flip-chip, evanescent coupler (reprinted with permission from ref. ^251^ ©Optica Publishing Group). In (**e**), embedded SOI transceivers in an organic substrate with optical fan-out using package level polymer waveguides and GSL patterned curved mirrors for optical coupling^382^ (©S. Suda et al., licensed under CC BY 4.0). In (**f**), a CPO design involving photonic and electronic die on different sides of a glass package substrate with optical connectivity using inter-chip couplers (reprinted from ref. ^131^, with the permission of AIP Publishing)

A scalable solution is to shift the fiber interface to the edge of the interposer or board where there is a larger available shoreline, and use integrated chip-to-interposer couplers to enable optical fan-out. This solution increases optical I/O density at the die level by over an order of magnitude and allows for the passive assembly via pick-and-place tools where electrical connections can be made simultaneously with a single bonding step^243^. The impact of such an architecture on switch package bandwidth and cost can be found in Fig. 16b alongside current scaling trends for pluggable and CPO-based systems. A variety of designs using inter-chip couplers in CPO systems have been proposed^9,58,59,131,152–154,244–251^. One such design is shown in Fig. 17a, b, where glass substrates with SiN_*x*_-to-SOI flip-chip evanescent couplers were proposed and prototyped with the goal of scaling optical I/O to >100 couplers/mm and total package data capacity to >1 Pbps^153^. A similar design is shown in Fig. 17c where a glass package substrate including TGVs and evanescent Si_3_N_4_-to-IOX couplers enabled optical and electrical fan-out to SMF arrays and board level solder bumps^154,247–249^. Architectures have also used inter-chip edge couplers to integrate III-V lasers on-chip and inter-chip evanescent couplers to optically fan-out from an SOI PIC to SMF arrays using a flexible polymer ribbon^9,244,246^. Others have used polymer waveguides on organic substrates with flip-chip evanescent couplers^251^ or organic interposers with embedded SOI die and free form couplers^58,59,250^ as shown in Fig. 17d, e, respectively. Separate proposed approaches make use of embedded glass die in organic substrates, creating photonic versions of the embedded bridges found in electronic 2.1D packaging architectures^252^. Layouts are also being explored for using 3D photonic and electronic packaging to connect photonic and electronic die on opposite surfaces of a glass core organic interposer, such as that in Fig. 17f^131^. Designs using high-performance Si interposers have been demonstrated as well, using evanescent couplers to connect thin Si_3_N_4_ interposer waveguides to thin Si_3_N_4_ PIC waveguides with simultaneous Au–Au bonding^253^.

While these examples illustrate significant possible improvements in scaling data capacity using inter-chip optical I/O, they also demonstrate three important trends: (1) the shift towards integrated waveguides at the package level, (2) the use of optical fan-out to accommodate pluggable fiber interfaces with large footprints, and (3) the critical need for substrates, such as glass, which provide higher performance than organics while enabling panel level processing. Additionally, studies into interposer-to-board couplers are necessary to provide a similar C-SWaP improvement to that seen in microelectronics when ball grid arrays with embedded board level Cu traces were implemented^28^.

### Biochemical sensing

**Fig. 18:**
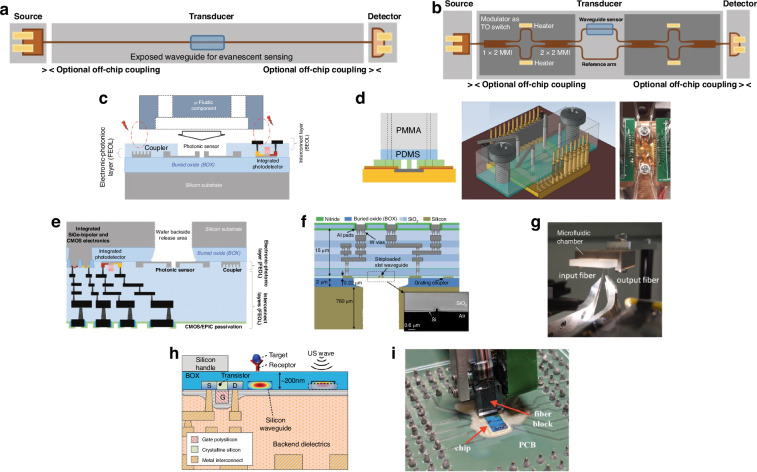
Examples of PIC based biochemical sensor packaging schemes where inter-chip couplers could lead to automated packaging. In (**a**) and (**b**), a simple evanescent sensor with sequential referencing and a sensor with integrated switches to enable accurate and time efficient referencing, respectively. The breaks in each figure represent breaks where inter-chip coupling would be required if source and/or detector were not fully integrated^257^ (©2024 C. Mitchell et al., licensed under CC BY 4.0). **c** A generalized top side accessible architecture is shown, assuming an integrated photodetector (reprinted from ref. ^258^, ©2022 A. Mai et al. with permission from Elsevier and licensed under CC BY-NC-ND 4.0). In (**d**), schematics and experimental images of a fully packaged active SiPh biosensor using a top side architecture (reprinted from ref. ^259^, ©2018 Elsevier B.V. with permission from Elsevier B.V.). In (**e**), a generalized back side accessible layout, assuming integrated photodetectors and electronics on the same die (reprinted from ref. ^258^, ©2022 A. Mai et al. with permission from Elsevier and licensed under CC BY-NC-ND 4.0). In (**f**), a cross section of the PIC including an integrated MRR sensor, photodetector, and grating coupler using the local backside etching technique^262^ (©2020 P. Steglich et al., licensed under CC BY 4.0). In (**g**), a fully packaged passive silicon biosensor using a back side layout (reprinted from ref. ^258^, ©2022 A. Mai et al. with permission from Elsevier and licensed under CC BY-NC-ND 4.0). **h**, **i** A partial back side etching architecture is shown alongside the fully packaged active silicon chip^263^ (©2021 C. Adamopoulos et al., licensed under CC BY 4.0)

The sensing of environmentally harmful biochemicals or deadly airborne diseases is a critical future application of PIC technology; however, the inability to cheaply package, reuse, or upgrade these systems has proved to be one of the challenges preventing their high volume manufacturing. In a fully integrated PIC based biochemical sensor, the light source, sensor, and detector are fabricated on the same die. The light source could be broadband with an optical filter, such as a superluminescent photodiode with a thermally tunable ring modulator. In a partially integrated sensor, the light source is often connected to the die using fiber connections with a grating coupler. The sensing element is commonly a passive resonator device such as an Mach-Zehnder interferometer (MZI) or micro-ring resonator (MRR), and the detector is integrated with the sensing and coupling element using a Ge photodetector. Considering common sources and photodetectors, especially in the mid-IR (2.5–25 μm)^254^, are not Si-CMOS or bipolar-CMOS (BiCMOS) foundry compatible, the fabrication of a fully integrated die followed by their quick disposal after use represents a costly cycle. Die need to be disposed of for several reasons including saturation of a functionalized die surface with bonded organic molecules (such as in refractometry)^255^; if a source, resonator, or detector fails; or if an upgrade is needed to detect different chemical signatures. It is also environmentally costly to regularly discard these PICs as active components contain precious metals which can then be expensive to reclaim via recycling processes^256^. This motivates the idea of separating the source or photodetector (or both) onto a different die from the passive sensing element, as shown for two common sensor types in Fig. 18a, b^257^. The connection between the source, or photodetector, and sensing element would then be made via an inter-chip coupler, allowing the passive element to be disposed of while maintaining the active components of the system. This solution and the choice of inter-chip coupler also depends on whether the sensing element is accessed via the top or the bottom, which will be described in more depth below.

To better understand how inter-chip connectors can decrease cost and improve functionality, we will describe state-of-the-art packaging and improvements that can be implemented going forward. Today, the most widely used approach is a top side accessible layout such that optical and electrical I/O, and access to the photonic sensing element, are accomplished on the device side of the die which is in a “face-up” orientation as shown in Fig. 18c^258^. In a top side accessible layout, fiber-to-chip connections are made using grating or edge couplers while electrical connections are made using wirebonds. The access to the sensing element is accomplished by doing a selective removal of the PIC cladding in the vicinity of the MZI or MRR and bonding a microfluidic chamber to flow the analyte over the surface of the sensing element. An example showing a top side accessible layout in practice is shown in Fig. 18d where a SiPh PIC has electrical connections made through a fan-out metal RDL, and optical connections using grating couplers. Because optical fibers typically cannot be permanently bonding to biosensing PICs due to their dispensable nature and high packaging cost^259^, only long-distance vertical optical coupling is possible in this top side accessible architecture. Therefore, the optical signal needs to traverse the entire fluidic gasket (~6 mm), requiring a fiber focuser to couple over a vertical distance of ~17 mm which resulted in additional losses of ~5 dB^259^. The usefulness of inter-chip coupling in this example is clear—polymer or PECVD waveguides in optical RDL layers could be patterned with evanescent, grating, or free form couplers to provide simultaneous electrical and optical fan-out away from the PIC. This could in turn allow for a reduction in PIC coupling losses, which approached 12 dB per connection in this study^259^.

Aside from the top side accessible packaging scheme, multiple backside accessible architectures have been proposed as well^258,260–263^. In a backside accessible design, as shown in Fig. 18e, the PIC substrate is etched to expose the sensing element to the analyte from the bottom. This allows for electrical and optical I/O to be connected on the die’s top side, which is then free to be flip-chip bonded. For a prototype, take the PIC layout shown in Fig. 18f, g where passive SOI MRRs were accessed via local backside etching^258,260–262^. By using a combination of a DRIE process to remove the bulk Si and a short wet etch to remove the SiO_2_ BOX, an opening through the back of the die was created. Similarly, Fig. 18h, i shows an electronic-photonic IC based biochemical sensor fabricated using GlobalFoundries 45 nm SOI CMOS process. This design can be applied either for label-free biosensing or ultrasound pressure induced sensing by removing the bulk Si handle and partially etching the BOX to tailor device sensitivity. In ref. ^264^, it was predicted that partially etching down to a 100 nm thick BOX would enhance intrinsic sensitivity by 7 times.

From the images in Fig. 18e, f, h, it can be seen how flip chip inter-chip couplers connecting to an optoelectronic interposer would facilitate multiple advantages. First, packaging cost would be decreased as passive assembly is enabled. Second, multiple die with different functionalized surfaces could be bonded, allowing for a single package to sense several different chemicals. Finally, assembling and bonding the passive sensing element by mechanical means versus permanent UV curable epoxies provides a path forward for pluggable die-to-interposer optical connections and the possibility for easy reuse, recycling, or upgrading.

### Photonic neuromorphic computing

**Fig. 19:**
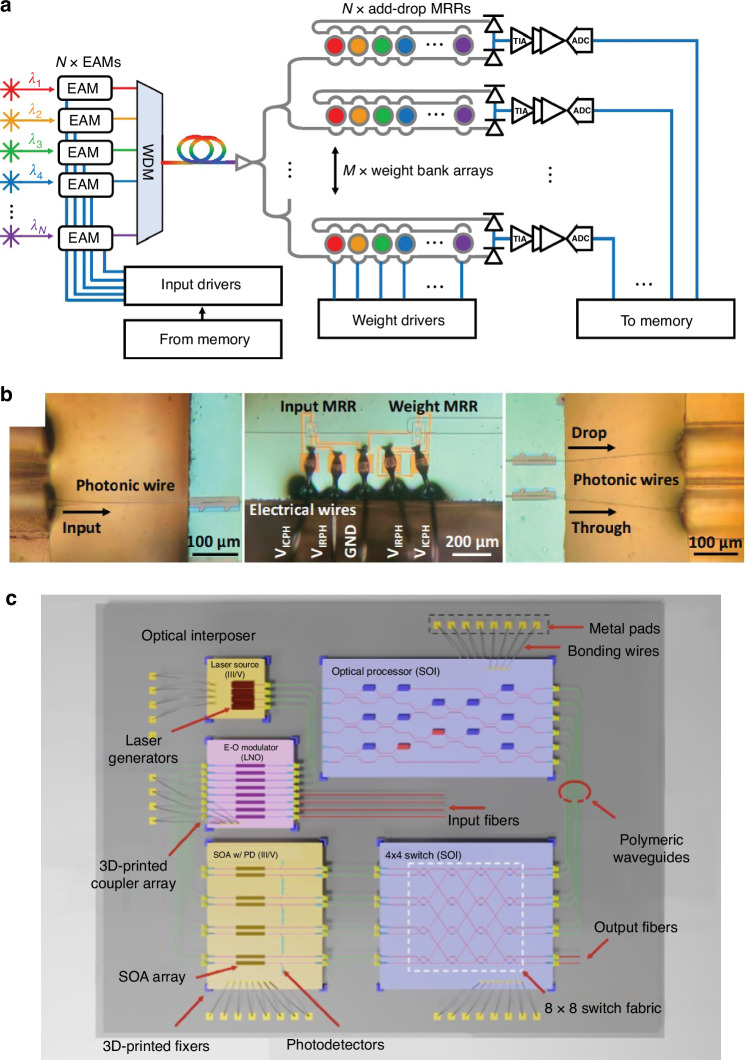
Examples of photonic computing architectures enabled by optical wirebonding, inter-chip couplers, and optical integration at the package level. **a**, **b** Example of a photonic computing unit using a multiwavelength architecture with a broadcast-and-weight scheme^265^ (©2023 Luan et al., licensed under CC BY 4.0). The layout in (**a**) shows how WDM is used to combine wavelengths into a single input channel which then splits into M lanes, each of which contains N modulators in the modulation and weight bank arrays which then terminate in M photodetectors. The top view in (**b**) shows the optical and electrical fan-out using PWBs and electrical wirebonds. **c** A schematic of a proposed photonic computing architecture where the lasers, optical processor, optical switch, and SOA are connected using polymer interposer waveguides and TPP printed micro reflectors^142^ (©2024 H. Huang, licensed under CC BY 4.0)

Optical computing provides the possibility of higher signal speed with lower propagation losses and less heat generation than electrical counterparts^265^. Because of this, the packaging of photonic processors with electrical logic and memory ASICs can enable superior performances for artificial intelligence applications^266^. There are two architectures used in photonic processors dependent on the light source: coherent or multiwavelength. Multiwavelength architectures use WDM with arrays of modulators, usually micro-ring modulators (MRMs), for optical processing. This is commonly implemented in a broadcast-and-weight scheme consisting of an input channel split into M modulation lanes and M weight lanes. Each modulation and weight lane has N modulators, each of which modulates a different wavelength. This layout is shown schematically in Fig. 19a. This type of scheme only requires one input channel (i.e., one SMF) which keeps coupling losses as a low contributor to the overall loss budget. With that said, three factors could drive photonic compute towards more advanced packaging interfaces: (1) the need for increased optical integration in a multichip layout including easy access to optical memory or switches, (2) the need for heterogeneous integration of SOAs to enable higher splitting powers and (3) the large numbers of electrical I/O used for drivers and collecting output signals from photodetectors could drive photonic compute systems towards flip-chip bonding. In these scenarios, an integrated flip-chip optical connector such as an evanescent or free form coupler could provide a potentially enabling technology for further integration and scaling.

As an example, take the photonic compute unit fabricated in ref. ^265^ which, instead of using one input channel with a splitter, uses 8 individual input channels without a splitter. Each input channel contains 9 operational wavelengths, demonstrating how an intensity modulated MRM based photonic tensor core could be used to decode a 28 × 28 pixel image. Each input channel contained 9 all-pass MRMs to form the modulation bank and 9 add-drop MRMs to form the weight bank. Each input channel also used a PWB to couple to a SMF, with each output channel terminating in a photodetector. The packaged processor is shown in Fig. 19b demonstrating the simultaneous electrical and photonic wirebonds present. The measured insertion loss due to the PWB interface was 7.5 dB/channel which was attributed to the etching process for the edge facet. For this prototype, which contains low connection volumes, photonic and electrical wirebonding provide simple, low cost solutions.

In terms of continued scaling, calculations for a theoretical upper limit show 578 operational wavelengths could be implemented with minimal crosstalk. A 512 × 512 (NxM) system was simulated to have a total loss budget of 60 dB assuming the fiber-to-chip connection contributed only 1.6 dB of loss. Having an array terminate in 512 photodetectors requires a higher level of electrical fan-out to connect to components in the package, a function flip chip assembly can enable. Moreover, a significant contributor to the loss budget was the decrease in optical power due to the splitter dividing power among 512 channels. Heterogeneous integration of an SOA in a photonic compute unit has been demonstrated using PWBs to address the power consumption challenge^267^. Further package level hybrid integration for photonic computing has been proposed using free form inter-chip couplers^142^. An example of such a package level layout is shown in Fig. 19c where interposer level polymer waveguides provide an optical RDL and are connected to lasers, modulators, switches, SOAs, and an optical processing unit using TPP based lenses^142^. Going forward, the limited compatibility of PWB technology with flip chip assembly indicates a different inter-chip connector may be needed. A high density inter-chip connector such as an evanescent or edge coupler could provide a path forward for cost-effective PIC assembly, SOA integration, and increased electrical fan-out, further highlighting the relevance of such couplers for future photonic compute systems.

### Optically connected memory

To increase the performance of DRAM stacks, two critical factors limiting the number of DRAM layers need to be addressed: thermal management and process node fabrication technology^268,269^. The first of these limitations, the thermal budget, dictates the thickness of the entire DRAM stack, while the process node used to fabricate each DRAM die controls the ability to make thinner die with higher channel counts. This is assuming the lateral or horizontal size of the DRAM die is fixed to avoid interconnect delay times. In terms of technology node, the 2021 Heterogeneous Integration Roadmap indicates that current stacking involves 50-μm-thick memory die, with thicknesses below 10 μm being investigated with bumpless wafer-on-wafer bonding and die thicknesses in the single micron range using advanced wafer thinning techniques^22^. As for thermal management, advancements in cooling technology including co-optimization of floorplanning^270^, vacuum or air gaps with chip-to-chip communication^271^, and a variety of microfluidic cooling designs^272,273^ demonstrate that 3D stacking of ultrathin DRAM die remains an optimistic approach moving forward.

**Fig. 20:**
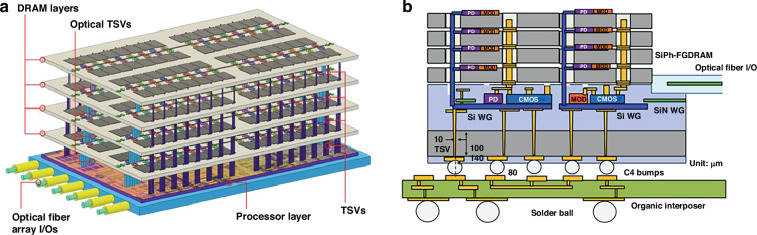
Proposed architectures showing how optical vias can enable optically connected memory. **a**, **b** Perspective and cross-sectional images showing photonic vias in silicon (along with evanescent intra-layer couplers and ULI fiber to Si_3_N_4_ couplers) implemented to accomplish 3D stacking of SiPh-FGDRAM dies with connections to a substrate level SiPh logic layer (reprinted with permission from ref. ^193^ ©2020 IEEE). In these images, the photodetector and modulator are located on the SiPh-FGDRAM die

Photonic via technology allows for optical connectivity between vertically stacked DRAM packages^268^. One such architecture is represented by Fig. 20a, b where electrical DRAM die are stacked with a Si photonic layer between every *n* DRAM die^268^. The Si photonic layer contains transceiver circuitry necessary to convert or encode the request for distribution to a DRAM die or back to the logical processing layer. Critically, each Si photonic layer is connected using a photonic via, specifically the wet etched 45° TIR mirror whose performance is outlined in Table 9. Such a design reduces the number of vertical through-die channels using bandwidth-dense optical links, thus minimizing the unused area in typical DRAM die, the area consumed by memory controllers, and the electrical power loss associated with longer distances^268^.

**Table 9:** Summary of photonic vias

Material	Substrate	Design	IL (dB)	1-dB Tolerance (μm)		BW (nm)	L × W (μm)	Process	Ref.
				Lateral	Via Length				
*Guided Mode Vias*									
Polymer to polymer	Glass	TIR mirrors	**<0.5**	± 2.5^a^ ± 5° (mirror *∠*)	/	/ (850)	50 (new)	New	^201^
SOI to SOI	Si	Grating & reflector	**1.3**	/	/	/ (1550)	13 × 5	Custom	^197^
SOI to a-Si	Si	TIR mirrors	8.4	< ± 0.12	<2	/ (1550)	0.5 × 0.8	Custom	^163^
**SOI to SOI**	**Si**	**Grating & reflector**	**0.96**	**± 2**	**± 0.13**^b^	**±10 (1550)**	**20** **×** **11**	**Custom**	^365^
**SOI to a-Si**	**Si**	**TIR mirrors**	**1.42**	/	**10**	/ **(1550)**	**0.3** **×** **1.9**	**Custom**	^366^
*Free Form Vias*									
Polymer to polymer lens	Glass	Mirror & lens	1.3^c^	**±16–19**^d^	**±210**^e^	/ (850)	230 (W)	Custom	^218,222^
Polymer to VCSEL/PD	Organic	Mirror & lens	1.6 to 3.3	/	/	/ (985)	35 (W)	New	^226^
		Mirror & lens	<1.5	± 75	/				^227–230^
	Organic & Si	Mirror & lens	2 to 2.5	± 40 (Rx) ± 25 (Tx)	/	/ (850)	150 (W)	New	^231^
IOX to VCSEL/PD	Glass	Mirror & lens	**1.73 to 4.32**	/	100	/ (1550)	254 (W)	New	^219,252,367^
Polymer to VCSEL/PD	Organic	Mirror & lens	1.6 to 2.9	± 12	/	/ (850)	45 (W)	New	^232^
Polymer to SOI	Glass	Mirror, lens, & grating	**0.62**^c^	± 3^d^ ± 4° (mirror *∠*)	/	/ (1550)	60 (W)	New	^208^
SOI to polymer	Si	Mirrors	**0.75**	± 1.5^f^	>200	/ (1310)	20 × 14	New	^205^
SOI to polymer	Organic	Grating, ball lens & mirror	3.4	± 14	/	± 10 (1310)	300 (W)	New	^206^
SOI to SOI lens	Si	Grating, reflector & lens	5.35^g^	± 2.5^g^	/	± 5 (1310)	275 (W)	Custom	^207^

The values in bold indicate that only simulation results were reported. Note that the “Material" column indicates the waveguide material followed by the material of the structure at the termination of the via

*BW* 1-dB bandwidth, / not reported

^a^Via to mirror

^b^BCB thickness tolerance

^c^Mirror only

^d^Lens to mirror

^e^Focal length tolerance

^f^Estimated based on MFD

^g^Grating to lens only

Similarly, 3D optically connected, stacked memory designs have been proposed where Si photonic fine-grain dynamic random access memory (SiPh-FGDRAM) die were stacked atop a Si photonic processor die^193^. Each SiPh-FGDRAM die contained integrated photodetectors and modulator devices with each die being optically connected using a 45° TIR mirror photonic via. By replacing electrical TSVs with photonic vias, latency and energy consumption were calculated to decrease by two fold while simultaneously decreasing footprint and improving data capacity through WDM^193^.

### Integrated quantum photonics

Integrated quantum photonics systems enable a variety of different capabilities including secure communication^274^ via quantum key distribution (QKD)^275^ or entanglement-distribution^276^, quantum photonic information processing^277^, and quantum physical and chemical simulators^278,279^. To build such devices, single photon sources, active reconfigurable devices, photon storage, low loss passive devices, wavelength converters, and single photon detectors^280^ need to be integrated or packaged together. One challenge is that each of these components could have its own material platform that suits it best. For example, a GaAs embedded quantum dot (QD) or diamond color center source may need to be interfaced with an SOI, Si_3_N_4_, or LiNbO_3_ modulator, followed by a niobium nitride (NbN) superconducting nanowire single photon detector (SNSPD)^281^ for optimal performance. Another roadblock is that different operating conditions may be necessary for different components, such as cryogenic temperatures of 4 K or below for single photon sources or SNSPDs^282^. While integration of SNSPDs with LiNbO_3_ Mach-Zehnder modulators (MZMs) and Si_3_N_4_ MEMS devices have been demonstrated at such temperatures^283,284^, the efficiency of the reconfigurable devices has also been shown to degrade in this thermal regime. This includes the Si_3_N_4_ or Si modulators reliant upon thermo-optic or free carrier plasma dispersion effects at room temperature^285,286^. Thus, within given quantum photonic package, components requiring cryogenic operating conditions may need to be connected via fiber or optical wirebond to components operating at room temperature. Such a configuration would also be useful for connecting atomic or ionic quantum devices for the formation of a modern quantum internet^287^.

**Fig. 21:**
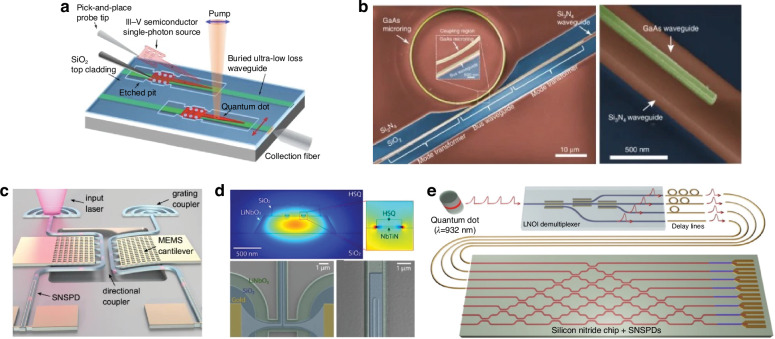
Examples of inter-chip couplers used for integrated quantum photonic applications. In (**a**), an evanescent coupler is used to connect a pick-and-place GaAs quantum emitter to ultra low loss Si_3_N_4_ passive circuitry^288^ (©2022 Chanana et al., licensed under CC BY 4.0). In (**b**), an evanescent coupler (right) is used to connect wafer bonded GaAs emitters and GaAs microring resonators (left) to Si_3_N_4_ passive circuitry^289^ (©2017 Davanco et al., licensed under CC BY 4.0). In (**c**), a reconfigurable Si_3_N_4_ circuit using MEMS devices is evanescently coupled to a NbTiN SNSPD^283^ (©2021 Gyger et al., licensed under CC BY 4.0). In (**d**), a cross sectional simulation (top) shows the material stack and coupling from LiNbO_3_ waveguides to NbTiN SNSPD and false colored SEM images show the integrated detector near Au pads (left) and a waveguide bend (right)^284^ (©2021 Lomonte et al., licensed under CC BY 4.0). In (**e**), a proposed hybrid integrated quantum photonic processor architecture in which QDs, LNOI modulators, Si_3_N_4_ passives, and SNSPDs are optically connected^292^ (reproduced with permission from SNCSC)

Inter-chip connectors introduce a solution to several of these packaging challenges such as the integration of different material platforms. For example, GaAs waveguides with embedded InAs QDs have been coupled with Si_3_N_4_ waveguides using a tungsten probe to pick and place the GaAs, as shown in Fig. 21a, b^288,289^. Submicron alignment was achieved with a triangular locking structures where the GaAS was cladded with SiO_2_ to secure it in position. Such a coupling concept was similarly used to connect InAs QDs embedded in InP waveguides to LiNbO_3_ waveguides using pick-and-place technology in ref. ^290^. Intra-chip evanescent couplers have also been used for such integration, such as in Fig. 21b where a GaAs layer was wafer bonded to the Si_3_N_4_ layer and patterned to form the evanescent coupler regions. These intra-chip evanescent couplers were also used to integrate GaAs MRRs which act as single photon sources whose QD radiative rate could be more precisely controlled. Similar devices and processes have also been used for integration of Si_3_N_4_ devices for four-wave-mixing-based wavelength conversion of single-photon lasers^291^.

Moreover, inter-chip couplers have also been able to integrate SNSPDs with reconfigurable devices at cryogenic temperatures, paving the way for feasible quantum state preparation and quantum logic at scale. In Fig. 21c, such an example is shown, where a niobium titanium nitride (NbTiN) SNSPD was integrated with a Si_3_N_4_ circuit containing MEMS cantilevers. The cantilever was used to modulate the incoming single-photon signal by changing the modal overlap as the cantilever arm changes position, and was demonstrated to operate at <100 mK^283^. Meanwhile, Fig. 21d shows a LiNbO_3_-on-insulator (LNOI) MZM integrated with a NbTiN SNSPD using evanescent coupling. Like the Si_3_N_4_ system, the LNOI system demonstrated bias-drift-free operation for over 12 h and high speed modulation for up to 1 GHz frequency while operating at temperature of around 1.3 K^284^.

At the system level, examples exist where the packaging of single photon sources, reconfigurable devices, low loss passive circuitry, and SNSPDs together is underway^292^. Such a system is shown in Fig. 21e, consisting of QDs coupled to LNOI reconfigurable devices, after which the signal is routed using optical fibers to low loss Si_3_N_4_ optical circuitry with integrated NbN SNSPDs. Inter-chip couplers are ideally suited for reducing packaging cost and improve system performance in this type of multi-PIC package. Specifically, the assembly cost and optical performance could be improved by integrating single photon sources and SNSPDs at the die level, and then flip-chip or photonic wirebonding the LNOI and Si_3_N_4_ PICs at the package level on an Si or glass substrate. The use of Si or glass interposers with inter-chip couplers in quantum photonic packaging is already underway^293,294^. For instance, glass interposers were used to connect a thick Si_3_N_4_ layer used for bright dissipative Kerr soliton generation with a thin Si_3_N_4_ layer used for passive circuitry^293^. While such an architecture would allow system designers flexibility in terms of die versus package level integration, further study is necessary to quantitatively assess the system level gains which can be achieved by their implementation.

### Heterogeneous integration of components for compact LiDAR systems

With the advent of autonomous vehicles and robotics, LiDAR has become an important application for PIC die. In essence, LiDAR requires the following building blocks: a laser source, an aperture to serve as an emitter and an aperture to serve as a receiver, and photodetectors to convert the reflected optical signal to an electrical signal for read out. In practice, additional components are necessary depending on what type of LiDAR is used. Two predominant LiDAR strategies involve frequency-modulated continuous wave (FMCW) LiDAR, a coherent form of LiDAR capable of simultaneously measuring distance and velocity, or time-of-flight (ToF) LiDAR, capable of measuring only distance but with a simpler overall design^295^. Both FMCW and ToF systems typically require optical amplifiers, such as Erbium doped fiber amplifiers (EDFAs), which can increase optical signal strength to >100 mW^296–298^. This additional power is also needed to overcome losses incurred by fiber-to-chip coupling and on-chip propagation^299^. Other needed components include electrical ICs for driving of active photonic devices, for readout, and for feedback control loops^298^.

Several of these functionalities can be miniaturized and improved by using on-chip Si photonic or III-V devices. This includes using an array of thermo-optic phase modulators and grating couplers as emitters in optical phased arrays (OPAs)^297,300–306^, Erbium doped Si_3_N_4_ waveguides^298^ or SOAs^299^ for amplification, and external cavity hybrid integrated lasers^298^ or monolithically integrated Erbium-lasers^307^ as optical sources. The current method of connecting separate components within these systems is with optical fibers, and thus they must contend with their packaging inefficiencies. The compactness of LiDAR systems is limited by fiber connections between components and by current architectures being limited to 2D layouts with large arrays of on-chip devices for unimpeded accessibility to the aperture. Currently, the emitters and receivers are also limited to either surface gratings (for HIC systems) or edge emitters (for LIC systems). Thus, flip-chip or photonic via devices would enable 3D architectures with a larger total emitter area, low loss integration of coherent sources, and emitter design flexibility through novel out-of-plane couplers.

**Fig. 22:**
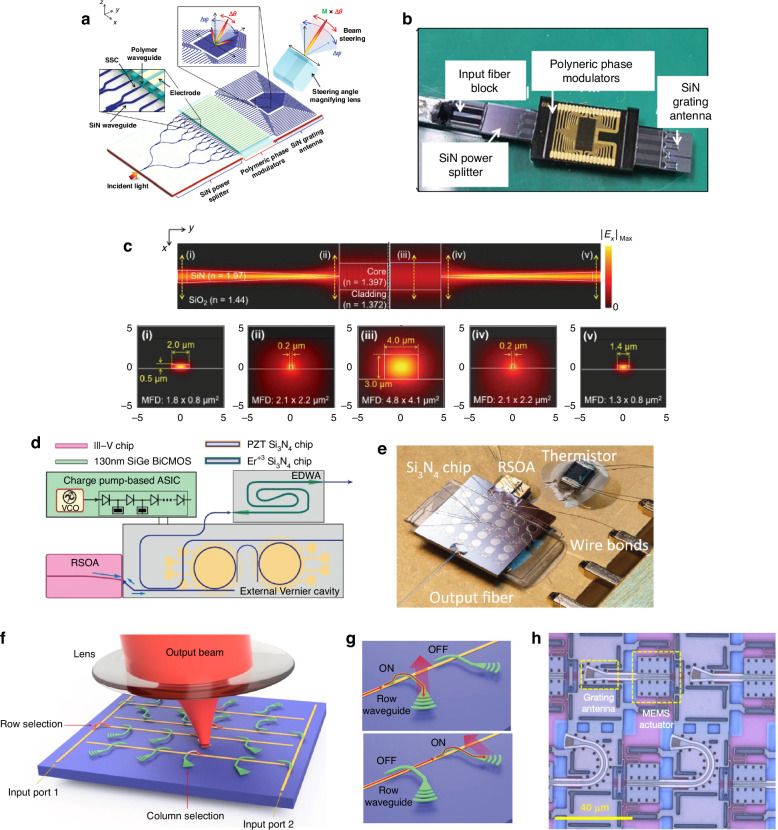
Examples of using inter-chip couplers and photonic vias in LiDAR systems. In (**a**), a diagram of a LiDAR system built by assembly of separate Si_3_N_4_ splitter and emitter die with a polymer thermo-optic phase shifter die using edge coupling, with the final packaged system shown in (**b**) and the transformation of the optical mode through the SiN to polymer edge coupler shown in (**c**) (reprinted with permission from ref. ^308^ ©2021 IEEE). In (**d**), a schematic showing a coherent LiDAR engine formed by edge coupling of a III-V reflective SOA die to a Si_3_N_4_ die with a Vernier cavity, with (**e**) showing the fully packaged structure^298^ (©2024 Lukashchuk et al., licensed under CC BY 4.0). In (**f**–**h**), schematics and a top-down microscope image showing MEMS based evanescent waveguide-to-waveguide couplers used as switches in an OPA unit^3^ (©2022 X. Zhang et al., licensed under CC BY 4.0)

There are several LiDAR examples where these types of couplers were used to integrate different material platforms. Edge couplers have been used for hybrid integration, as shown in Fig. 22a–c, and evanescent couplers have been used for monolithic integration of polymer-based phase modulators in an edge emitting PIC based ToF LiDAR setup^308,309^. Evanescent couplers were also used for integrating SOI-based thermo-optic phase shifters with Si_3_N_4_ passive circuitry to take advantage of SOI’s relatively high thermo-optic coefficient (1.86 × 10^−4^, ref. ^310^)^311^. The use of the polymer-based materials for phase shifting allowed for higher thermo-optic coefficient (−6 × 10^−5^ for the polyimide core^312^ versus 2.45 × 10^−5^ K^−1^ in Si_3_N_4_)^310^ with high confinement in Si_3_N_4_ which does not suffer from the same two-photon absorption-related issues at high optical powers (*β*_TPA_ ≈ 9 × 10^−12^ m/W for SOI versus 0 m/W for Si_3_N_4_)^310^. Similarly, edge couplers have been used to connect a III-V reflective SOA die to a Si_3_N_4_ die containing a Vernier ring as shown schematically in Fig. 22d and experimentally in Fig. 22e, thus creating a coherent external hybrid cavity laser^298^. The output from the Si_3_N_4_ die was then fiber connected to a separate Er doped Si_3_N_4_ waveguide chip which provided amplification prior to a free space emitter. Further iterations could be improved by using flip-chip assembly of the external cavity laser with the Er doped Si_3_N_4_ waveguide chip and/or a SiPh OPA die in place of the free space emitter.

As mentioned above, inter-chip couplers and photonic vias also offer 3D OPA architectures. Today, the thermo-optic modulator and the grating coupler arrays are side by side in a 2D layout because the emitter section needs to be accessible. One solution is to design the LiDAR package such that the emitter layer is facing up while the active devices are underneath it. This design allows for electrical and optical connections to be located lower in the stack, with the connection between the two layers enabled through electrical and optical vias. By doing this, OPA element pitches <1 μm can be achieved^198^. A prototype of such a design has been demonstrated, where input light was split into 120 channels containing thermal phase modulators and 2-μm pitch gratings^198^. The splitting and modulation occurred in a separate layer from the grating emitters and the two layers were connected using the offset 45° TIR mirrors. The densely packed OPA design realized emitting areas with very large fill factors (~95%) while reducing die footprint through the 3D layout. The prototype was one aspect of a unit cell which included an ASIC underneath the emitting and spitting/modulation layers to control the modulators^193^. This unit cell can then be bonded to an interposer, with electrical coupling done using solder arrays and optical coupling done using 3D ULI waveguides.

While the discussion thus far has been focused on OPA based LiDAR, waveguide-to-waveguide couplers have also been used to enable focal plane switch array (FPSA) LiDAR as well^3^. Unlike OPA based systems which require precise phase and amplitude control for each emitter, FPSA systems only require an array of emitters, each with a switch mechanism, and a single lens covering all the emitters to enable beam steering. Waveguide couplers with high efficiency, but relatively low alignment tolerance, make excellent switches which can reduce the C-SWaP and increase the pixel count to enable higher performance. For example, evanescent couplers with MEMS devices have been implemented as switches in compact FPSAs, as shown in Fig. 22f–h^3^. Using these switches, a LiDAR system with 16,384 pixels (32 times larger than previous records) was demonstrated with a wide field of view (FoV, 70° × 70°), a fine addressing resolution (0. 6° × 0. 6°), a narrow beam divergence (0.050° × 0.049°) and a random-access beam addressing with sub-MHz operation speed^3^. Though further experiments are necessary to bring the described technologies to industrial LiDAR systems, they highlight the potential of photonic couplers to improve C-SWaP by enabling functions such as coherent source integration, micron scale OPA emitter pitches, and order of magnitude larger FPSA pixel counts.

## Conclusion

In retrospect, this paper discussed the inter-chip and intra-chip coupling of light using edge, grating, evanescent, free form, and cantilever couplers along with optical wirebonding technology. Methods for coupling of light through PICs using guided mode or free form vias was discussed in the context of different substrate materials including silicon, glass, and organics. The current and future use of these couplers in relevant applications including datacenter switch packages, biochemical sensors, optically connected memories, optical computing, integrated quantum photonics, and LiDAR systems was discussed. There are a few takeaways from the presented data which demonstrate the critical role these technologies will play in integrated photonic systems.

First, the inter-chip or flip chip optical couplers discussed here enable the elimination of fiber-to-chip interfaces and the manufacturing challenges and inefficiencies associated with those interfaces. This is important in two ways: connection density (i.e., total bandwidth) and cost. In terms of connection density, the inter-chip optical couplers break through the 8 connections per mm barrier imposed by the standard 125 μm cladding of SMF arrays, allowing for connection densities greater than 100 connections per mm—over an order of magnitude increase. The ability to scale the number of connections to the photonic package in parallel provides another vector for increasing the total package bandwidth along with the scaling of wavelengths per channel, polarizations per channel, bit rate per wavelength, and optical modes per channel. The other effect is on the cost of manufacturing photonic packages—inserting an inter-chip optical coupler in place of a fiber-to-chip interface allows for passive using automated pick-and-place tools instead of active alignment and assembly, which can increase throughput and decrease cost.

A second takeaway is understanding the role each inter-chip coupling type may play in the hierarchies of future photonic systems, as each type has substantial tradeoffs evidenced by Fig. 6. For material integration, chip-to-chip, chip-to-RDL, or even chip-to-interposer connections, where the connection density will need to be the highest and the electrical bumps are thinnest, evanescent couplers offer an optimized solution owing to their tight pitches (<10 μm), wide wavelength window (>100 nm), low coupling loss (<1 dB), and location away from the edge facet for simpler packaging and assembly. With designs using multisegmented tapers which increase alignment tolerance to greater than 1–2 μm such that passive assembly can be used, the remaining downside is in the taper length and need for BEOL customization. Because the length is needed in part to achieve a wider alignment tolerance, the length problem will be resolved as pick-and-place tools continue to improve alignment accuracy, such that the taper length can be decreased down to the shortest length required for adiabatic coupling (typically <100 μm for Si based devices). The BEOL customization can also be overcome by using multiple layers, for example SiN for SiPh die, such that the transfer through the BEOL layers happens over several transitions. In light of this, evanescent couplers can be seen as the photonic analogy to direct Cu–Cu bonding in electrical packaging. In fact, when combined with hybrid bonding, the evanescent coupler offers the highest electronic-photonic connection density possible.

For interposer-to-substrate or substrate-to-board connectivity, free form couplers are an excellent fit—the wide allowable vertical coupling gaps (20 μm to >700 μm) allows for simultaneous integration of electrical μm-bumps while maintaining broadband, alignment tolerant operation with tighter pitches than SMF arrays. The aspect to be addressed with free form couplers is in their fabrication—they often require serial fabrication techniques which incorporated new materials or processes compared to standard CMOS process flows. Since the three primary processes used are dicing, TPP, and GSL, there is the need to parallelize these processes by continuing to develop hot embossing^313^ or NIL^314^, the latter of which has already been used to create 3D photonic devices^137^ and is in consideration for use in high volume semiconductor manufacturing^315^. Once the signal is on the package substrate or board, similar free form couplers can be implemented for pluggable fiber-to-board connectors^316–319^, allowable for effective optical fan-out in an identical way to electrical fan-out.

As described above, not all applications require significant connection volumes, but these applications still require low cost optical interfaces. It is for these applications that optical wirebonding techniques are likely to play the biggest role due to their versatility in overcoming highly custom packaging requirements. Of the technologies presented, PWBs and 3D ULI will likely become the most dominant. While PWBs are already offered commercially with automated design capabilities in the manufacturing flow^320^, 3D ULI represents a substrate agnostic method for creating fully embedded connections in glass based materials. What is required for both is not necessarily a method for parallelizing fabrication (albeit useful), it is to decrease the write time for such connections to be in line with those of automated (or even manual) electrical wirebonding equipment (<0.1 seconds per connection). Current techniques are roughly 2–3 orders of magnitude away from this marker, preventing their ubiquitous use due to high costs per connection. With that said, although the processes themselves are new to CMOS process flows, PWBs and 3D ULI have been fabricated on foundry made die without altering FEOL processes or raising temperatures above BEOL or FEOL thermal budgets—an excellent start towards demonstrating full foundry compatibility.

The third takeaway from this review is that photonic vias remain at the research and development scale and represent a largely unexplored space, but one which will may prove critical to photonic integration at the die, package, and board level. At the package and board level, photonic vias will need to play a role similar to the cores in organic package substrates or TGVs and TSVs in glass or silicon interposers, respectively, which work in tandem with solder and Cu pillar bumps to allow for electrical signals to vertically fan-out to the board. In other words, photonic vias will likely have to work together with inter-chip optical couplers to achieve full system level photonic integration. The vias used at this scale would likely be free form vias with transparent substrates (glass) or through hole etched organic materials. At the chip scale, photonic vias could allow for access to the FEOL waveguide layer without having access to the top or BEOL layers on the die, such as in biochemical sensing or LiDAR applications. Perhaps more importantly, compact guided mode photonic vias may present the opportunity to obtain optically connected electronic devices all the way down to the device level. Here, it was presented that this could be useful for optically connected memory between stacked DRAM die. And yet, another speculative use case would be using less than 5-μm-thick photonic vias to achieve attajoule scale (10^−18^ J/bit) optical connectivity by connecting Ge based photodetectors at the transistor scale in a monolithic integration scheme as suggested by Miller^321^.

Thus, overall given the immense research in 3D photonic packaging, we conclude inter-chip couplers (i.e., optical bumps) and intra-chip couplers (i.e., photonic vias) have a similar potential to scale cost and performance in PIC packaging as the advent of the electrical solder bump or electrical via had in electronics packaging.

## Data Availability

All data are available from the corresponding authors upon reasonable request.
